# Pazopanib and Trametinib as a Synergistic Strategy against Osteosarcoma: Preclinical Activity and Molecular Insights

**DOI:** 10.3390/cancers12061519

**Published:** 2020-06-10

**Authors:** Giulia Chiabotto, Giovanni Grignani, Maja Todorovic, Valentina Martin, Maria Laura Centomo, Elisa Prola, Giorgia Giordano, Alessandra Merlini, Umberto Miglio, Enrico Berrino, Lucia Napione, Claudio Isella, Federica Capozzi, Marco Basiricò, Cristina Marsero, Ilaria Gerardi, Tiziana Venesio, Dario Sangiolo, Massimo Aglietta, Lorenzo D’Ambrosio, Ymera Pignochino

**Affiliations:** 1 Division of Medical Oncology, Candiolo Cancer Institute, FPO-IRCCS, Str. Prov. 142 km 3.95, 10060 Candiolo (TO), Italy; giulia.chiabotto@unito.it (G.C.); giovanni.grignani@ircc.it (G.G.); maja.todorovic@gmail.com (M.T.); valentina.martin@gmail.com (V.M.); marialaura.centomo@ircc.it (M.L.C.); elisa.prola447@edu.unito.it (E.P.); giorgia.giordano18@gmail.com (G.G.); alessandra.merlini@ircc.it (A.M.); federica.capozzi@ircc.it (F.C.); marco.basirico@ircc.it (M.B.); cristina.marsero@gmail.com (C.M.); ilaria.gerardi91@gmail.com (I.G.); dario.sangiolo@ircc.it (D.S.); massimo.aglietta@ircc.it (M.A.); lorenzo.dambrosio.md@gmail.com (L.D.); 2 Department of Oncology, University of Torino, 10124 Torino, Italy; 3 Unit of Pathology, Candiolo Cancer Institute, FPO-IRCCS, 10060 Candiolo (TO), Italy; umberto.miglio@ircc.it (U.M.); enrico.berrino@ircc.it (E.B.); tiziana.venesio@ircc.it (T.V.); 4 Department of Medical Sciences, University of Torino, 10100 Torino, Italy; 5 Laboratory of Vascular Oncology, Candiolo Cancer Institute, FPO-IRCCS, 10060 Candiolo (TO), Italy; lucia.napione@polito.it; 6 Department of Applied Science and Technology, Politecnico di Torino, 10060 Torino, Italy; 7 Laboratory of Oncogenomics, Candiolo Cancer Institute, FPO-IRCCS, 10060 Candiolo (TO), Italy; claudio.isella@ircc.it

**Keywords:** osteosarcoma, pazopanib, trametinib, tyrosine-kinase inhibitors, MEK6, EphA2, IL-7R

## Abstract

Receptor tyrosine kinases (RTKs) inhibitors’ activity in advanced osteosarcoma is significant but short-lived. To prevent or at least delay drug resistance, we explored a vertical inhibition by combining drugs acting at different levels of the RTK pathways (pazopanib + trametinib). We studied pazopanib + trametinib antitumor activity both in vitro and in vivo (MNNG-HOS and KHOS xenografts in NOD/SCID mice) investigating the molecular mechanisms and potential escapes. The involvement of MAPK-PI3K pathways was validated by Nanostring technology, western blot and by silencing/overexpression experiments. Pazopanib targets were expressed on seven osteosarcoma cell lines and their pathways were activated. Pazopanib + trametinib exhibited synergistic antitumor activity by inducing apoptosis and inhibiting ERK1/2 and Akt. In vivo antitumor activity was shown in osteosarcoma-bearing mice. The drug combination significantly down-modulated RTK Ephrin Type-A Receptor 2 (EphA2) and Interleukin-7 Receptor (IL-7R), whereas induced mitogen-activated protein-kinase kinase (MAPKK) MEK6. EphA2 silencing significantly reduced osteosarcoma cell proliferation and migration, while impeding MEK6 up-regulation in the treated cells significantly increased the antitumor effect of the studied drugs. Moreover, the up-regulation of MEK6 reduced combination activity. Pazopanib + trametinib demonstrated synergistic antitumor effects in osteosarcoma models through ERK and Akt inhibition and EphA2 and IL-7R down-modulation. MEK6 up-regulation might evoke escaping mechanism.

## 1. Introduction

Osteosarcoma is the most common primary bone tumor in children and young adults. Despite the fact the outcomes of localized disease are improved by chemotherapy, in the presence of unresectable relapses or metastatic spread prognosis remains poor. Receptor tyrosine kinases (RTKs), such as vascular endothelial growth factor receptors (VEGFRs), fibroblastic growth factor receptors (FGFRs), and platelet-derived growth factor receptors (PDGFRs) are each involved in osteosarcoma progression [1,2,3]. In the last decade, tyrosine-kinase inhibitors (TKIs) targeting osteosarcoma oncogenic pathways have been explored to improve the limits of chemotherapy in advanced settings [4,5,6,7,8,9]. The clinical activity of TKIs was significant but short-lived, due to primary or acquired resistance related to activation of RTK downstream pathways, among which Ras-Raf-MEK-ERK and PI3K/Akt were the most commonly involved [10,11,12]. Our previous data have shown inhibition of the PI3K-Akt-mTOR pathway improved TKI activity, but the problem of acquired resistance persisted [13,14]. In this paper, we pursued the vertical inhibition of RTK pathways acting at different levels: upstream with pazopanib [15,16], targeting main RTKs involved in osteosarcoma (PDGFRs, VEGFRs, FGFRs) and downstream with trametinib, a MEK1/2 specific inhibitor [17,18,19,20]. Moreover, we chose this combination for its good tolerability and the more favorable toxicity profile of pazopanib compared with other TKIs that frequently require significant dose reduction when combined with other drugs [21,22,23,24]. With this strategy we obtained a synergistic antitumor activity against several osteosarcoma models and the complete inhibition of both Ras-Raf-MEK-ERK and PI3K/Akt pathways. Moreover, deeply investigating the molecular perturbation evoked by pazopanib and trametinib combination, we identified EphA2, IL-7R, and MEK6 as potential novel targets in osteosarcoma.

## 2. Results

### 2.1. The Combination of Pazopanib and Trametinib Inhibits PI3K/Akt and MEK/ERK Pathways in Osteosarcoma

**Figure 1:**
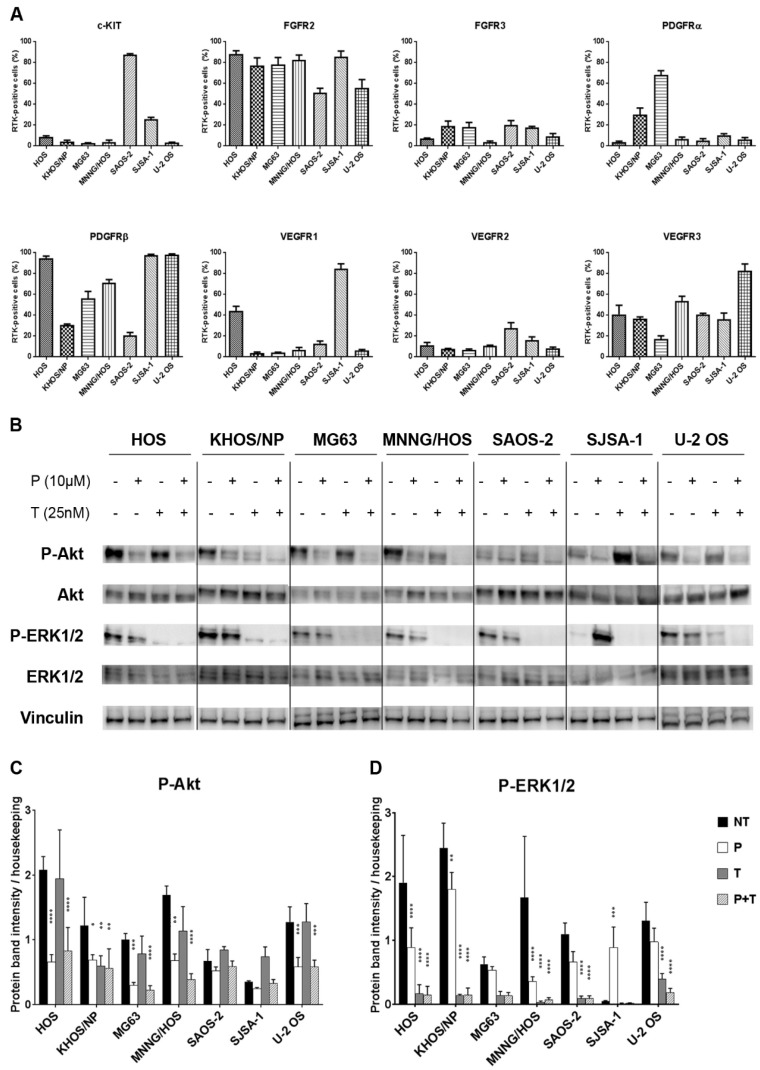
The combination of pazopanib and trametinib modulated MAPK and PI3K/Akt pathways in seven osteosarcoma cell lines. (**A**), Flow cytometry analysis of RTKs expression in seven osteosarcoma cell lines. (**B**), Representative western blot analysis of P-Akt (Ser473), P-ERK1/2 (Thr202/Tyr204), Akt, and ERK1/2 at basal conditions and after 24 h of treatment with pazopanib, trametinib, and their combination. Vinculin expression was evaluated as a loading control. (**C**,**D**), Densitometric analysis of protein band intensity of P-Akt (**C**) and P-ERK1/2 (**D**) normalized to housekeeping bands. Data are expressed as mean ± SD. Independent experiments were performed three times in seven osteosarcoma cell lines. * *p* < 0.05, ** *p* < 0.01, *** *p* < 0.001, **** *p* < 0.0001 vs. controls. NT = not treated, P = pazopanib, T = trametinib, P + T = pazopanib + trametinib combination.

Pazopanib molecular targets (c-KIT-CD117, FGFR2-3 PDGFRα-β, VEGFR-1-2, and -3) displayed a plasma membrane heterogeneous degree of expression in seven osteosarcoma cell lines (Figure 1A), with FGFR2, VEGFR3, and PDGFRβ represented most. Western blot analyses showed activation of main downstream signaling pathways (PI3K/Akt/mTOR and Ras/Raf/MEK/ERK) of expressed RTKs in all osteosarcoma cell lines (Figure 1B–D).

Since both pazopanib and trametinib targets were found expressed and activated in osteosarcoma cells, we monitored their actual modulation by western blot analysis following 24 h of incubation with 10 µM pazopanib and 25 nM trametinib, either alone or in combination. These treatments had no effect on Akt or ERK1/2 expression, but reduced their phosphorylation (Figure 1B–D). In particular, compared to the untreated control, pazopanib significantly reduced Akt phosphorylation in six of seven osteosarcoma cell lines (HOS, KHOS/NP, MG63, MNNG/HOS, SJSA-1, and U-2 OS); whereas only a slight decrease was obtained in ERK1/2 phosphorylation in five of seven cell lines (HOS, MG63, MNNG/HOS, SAOS-2, and U-2 OS). On the contrary, trametinib induced a significant reduction of Akt phosphorylation in KHOS/NP only (Figure 1B,C) and ERK1/2 was completely dephosphorylated in all osteosarcoma cell lines (Figure 1B,D). Interestingly, the drug combination strongly reduced Akt phosphorylation and completely inhibited ERK phosphorylation in all cell lines tested (Figure 1B–D).

### 2.2. Antitumor Activity of Pazopanib and Trametinib Combination against In Vitro and In Vivo Osteosarcoma Models

Given the effective inhibition of PI3K/Akt/mTOR and Ras/Raf/MEK/ERK pathways, we explored pazopanib and trametinib antitumor activity in osteosarcoma preclinical models. By means of cell viability assays, we determined the IC50 and the combination index after 72 h of treatment. As monotherapies, pazopanib and trametinib showed an antiproliferative effect against six of seven (HOS, KHOS/NP, MG63, MNNG/HOS, SJSA-1, and U-2 OS) and five of seven (KHOS/NP, MG63, MNNG/HOS, SAOS-2, and SJSA-1) treated osteosarcoma cell lines, respectively. Notably, SJSA-1 was remarkably sensitive to both single agents. The combination in all other cell lines (HOS, KHOS/NP, MG63, MNNG/HOS, SAOS-2, and U-2 OS) was highly synergistic even overcoming the resistance to pazopanib in SAOS-2 cells and to trametinib in HOS and U-2OS cells (Table 1).

**Figure 2:**
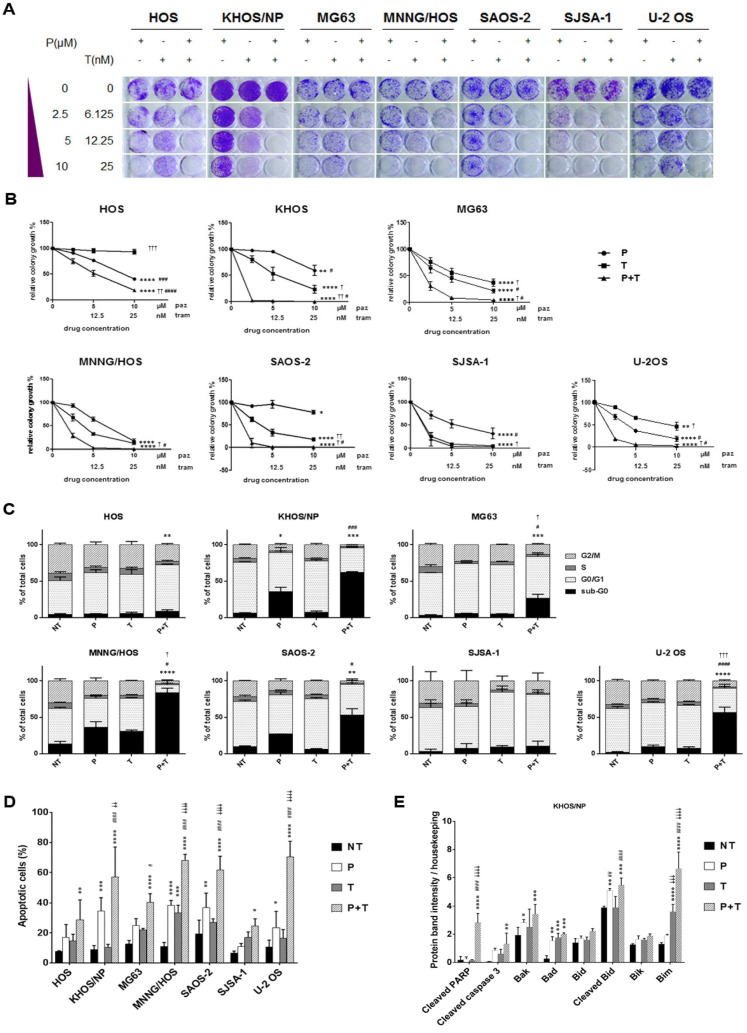

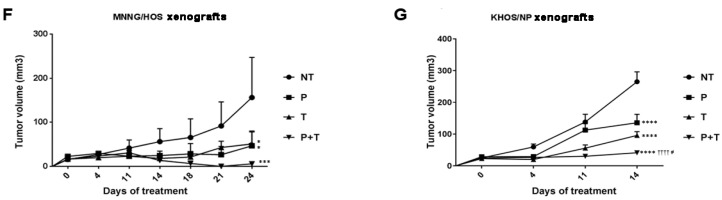
Antitumor effect of the combination of pazopanib and trametinib in vitro and in vivo. (**A**) Representative images of colony-forming assay on osteosarcoma cell lines, treated with scalar doses of pazopanib (0–10 µM), trametinib (0–25 nM), and their combination for 7 days. (**B**), Quantification of colony growth in treated cells compared to control cells. (**C**,**D**), Percentage quantification of flow cytometry analysis of (**C**) DNA content (cell cycle distribution) and (**D**) Annexin V-PI staining (apoptosis) in osteosarcoma cells after 72 h of treatment with pazopanib (10 µM) and trametinib (25 nM) as single agents and in combination. (**E**) Densitometric analysis of pro-apoptosis Bcl-2 family protein expression normalized to housekeeping bands. Data are expressed as mean ± SD. Independent experiments were performed three times in KHOS cells. (**F**,**G**), in vivo antitumor effect of pazopanib and trametinib in human osteosarcoma xenograft models. After tumor establishment, six mice per group were randomized to receive pazopanib (40 mg/kg, 5 days/week), trametinib (0.1 mg/kg, 5 days/week), their combination, or vehicle alone (control group) daily. Tumor volume is reported as mean  ±  SD. * *p* < 0.05, ** *p* < 0.01, *** *p* < 0.001, **** *p* < 0.0001 vs. controls; # *p* < 0.05, ## *p* < 0.01, ### *p* < 0.001, #### *p* < 0.0001 vs. trametinib; † *p* < 0.05, †† *p* < 0.01, ††† *p* < 0.001, †††† *p* < 0.0001 vs. pazopanib. NT = not treated, P = pazopanib, T = trametinib, P + T = pazopanib + trametinib combination.

The antiproliferative effect of pazopanib, trametinib, and their combination was further validated by colony growth assays. After 7 days of treatment, pazopanib + trametinib combination significantly reduced osteosarcoma cell colony growth when compared to single agents in all cell lines, but for SJSA-1 cells which were highly sensitive to single agents (Figure 2A,B)

Next, we investigated whether the antiproliferative effect of the combination of pazopanib and trametinib was related to impairment of cell cycle progression. The combination of the two drugs significantly reduced the percentage of proliferating cells (phase G_2_/M) by blocking the cell cycle in the G_0_/G_1_ phase and increasing the percentage of apoptotic and dead cells (sub-G_0_ phase) compared to both single agents (in three of seven osteosarcoma cell lines) and untreated controls (in six of seven tested cell lines, Figure 2C).

We further confirmed the induction of apoptosis by Annexin V and PI staining. Indeed, the combination of pazopanib and trametinib significantly increased the proportion of apoptotic cells compared to both single agents (in four of seven osteosarcoma cell lines), and untreated controls (in all osteosarcoma cell lines, Figure 2D and Figure S1). To deepen into apoptosis triggering, we investigated the expression of the pro-apoptotic Bcl-2 family members and cleavage of caspase 3 and PARP. We observed that pazopanib and trametinib combination stimulated the expression of pro-apoptotic proteins Bad, Bim, Bak, Bik, Bid and the consequent cleavage of caspase 3 and PARP in KHOS cells (Figure 2E and Figure S2).

Based on these promising results, we investigated the antitumor effect of pazopanib, trametinib, and their combination in osteosarcoma MNNG/HOS and KHOS xenograft models. At the end of the experiments (24 and 14 days of treatment for MNNG/HOS and KHOS xenografted mice, respectively), both drugs induced a significant inhibition of tumor growth compared to the untreated controls (MMNG-HOS, *p* < 0.05; KHOS, *p* < 0.0001). The combination did so to an even greater extent (MMNG-HOS, *p* < 0.001 vs. untreated; *p* < 0.05 vs. single agents, Figure 2F; KHOS, *p* < 0.0001 vs. untreated and pazopanib; *p* < 0.05 vs. trametinib, Figure 2G). At the end of the experiment, no weight loss was registered in all the groups of treatment and no signs of toxicity was observed after gross necropsy.

### 2.3. Pazopanib and Trametinib Combination Modulates Key Transducers of MAPK and PI3K Pathways

**Figure 3:**
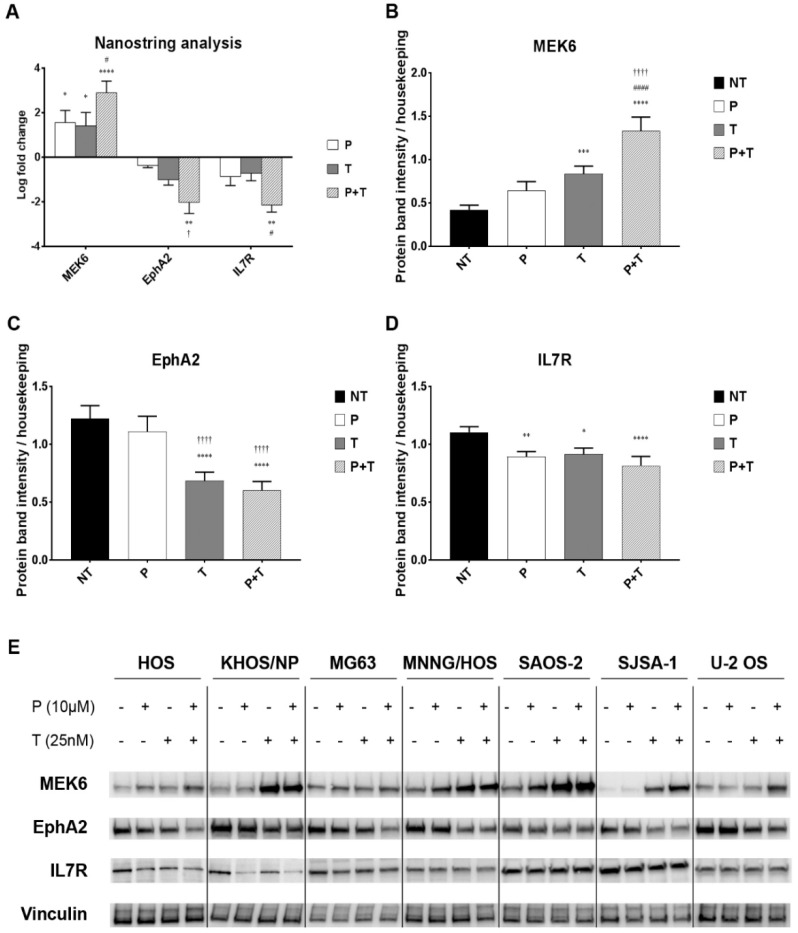
The combination of pazopanib and trametinib modulated MEK6, EphA2, and IL7R at mRNA and protein level. (**A**), Average mRNA log-fold change in pazopanib (P), trametinib (T) and combination (P + T) treated U2-OS, KHOS, and MNNG-HOS cells analyzed by nCounter^®^Vantage 3D™RNA MAPK-PI3K Pathways Panel with a Nanostring instrument and nSolver software. (**B**–**D**), Densitometric analysis of protein band intensity normalized to housekeeping bands (average expression in seven cell lines). (**E**), Representative western blot in seven cell lines. Data are expressed as mean ± SEM. * *p* < 0.05, ** *p* < 0.01, *** *p* < 0.001, **** *p* < 0.0001 vs. controls; # *p* < 0.05, ## *p* < 0.01, ### *p* < 0.001, #### *p* < 0.0001 vs. trametinib; † *p* < 0.05, †† *p* < 0.01, ††† *p* < 0.001, †††† *p* < 0.0001 vs. pazopanib. NT = not treated, P = pazopanib, T = trametinib, P + T = pazopanib + trametinib combination.

To delve deeply into the molecular mechanisms behind pazopanib and trametinib combination activity, we performed a high-throughput screening of transcriptional modifications of 380 MAPK and PI3K-pathway-related genes on three osteosarcoma cell lines (HOS, MNNG/HOS, U-2 OS) with the NanoString^®^ nCounter Technology. In all tested cell lines, the combination of pazopanib and trametinib significantly induced the expression of the MEK6 gene (log-fold change = 2.90 ± 0.51) when compared to untreated controls (*p* < 0.0001) and to trametinib-treated cells (Figure 3A, *p* < 0.05). Moreover, the drug combination significantly reduced the expression of EphA2 gene (log-fold change = −2.02 ± 0.50) when compared to the untreated controls (*p* < 0.01) and to pazopanib-treated cells (*p* < 0.05). Also the expression of IL7R gene was significantly reduced by the combination (log-fold change = −2.15 ± 0.31) when compared to untreated controls (*p* < 0.01) and to trametinib-treated cells (*p* < 0.05, Figure 3A).

To validate these findings, we checked MEK6, EphA2, and IL7R also at protein level in all seven osteosarcoma cell lines after 24 h of treatment with pazopanib (10 µM), trametinib (25 nM), and their combination. Consistently with the previous experiments, we demonstrated that the two-drug combination significantly upregulated MEK6 protein expression (*p* < 0.0001), and downregulated EphA2 (*p* < 0.0001) and IL-7R (*p* < 0.001) compared to untreated controls (Figure 3B–E). We did not observe any change in p38 expression and phosphorylation (Figure S3).

### 2.4. EphA2 Involvement in Osteosarcoma Cell Proliferation and Migration

**Figure 4:**
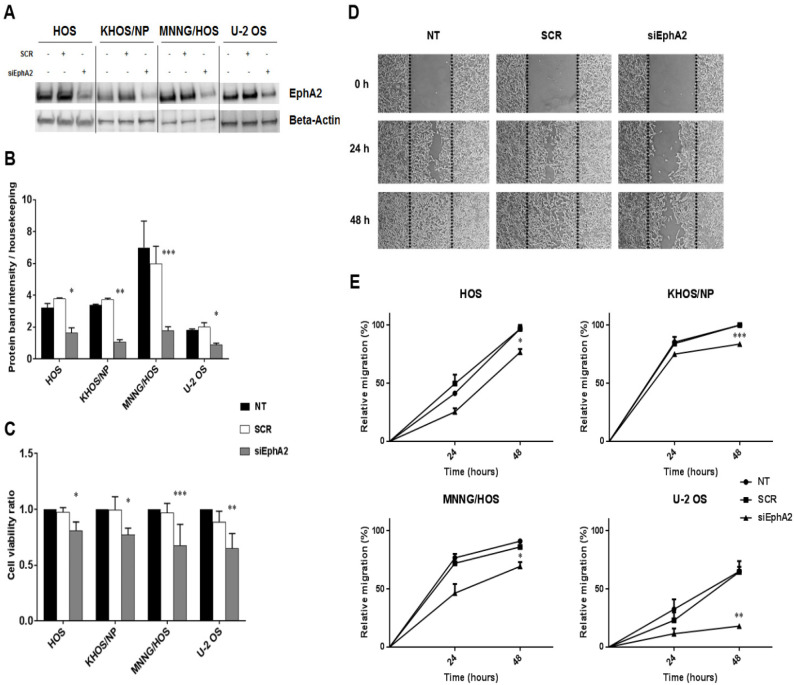
Down-regulation of EphA2 decreased osteosarcoma cell viability and migration. (**A**), Western blot analysis of EphA2 protein expression in four osteosarcoma cell lines (HOS, KHOS/NP, MNNG/HOS and U-2OS) after 24 h-transfection with specific siRNA against EphA2. β-actin expression was evaluated as loading control. (**B**), Densitometric analysis of protein band intensity of EphA2 normalized to housekeeping bands. (**C**), Average cell viability measured in four osteosarcoma cell lines after 72 h of transfection with specific siRNA against EphA2 and normalized to average cell viability of untreated cells. (**D**), Representative photomicrographs of wound healing assay on EphA2-silenced MNNG/HOS cells at 0, 24, and 48 h post-scratching. Black dotted lines indicate wound borders at the beginning of the assay. Original magnification: ×50. (**E**)**.** Percentage of relative cell migration of EphA2-silenced osteosarcoma cells, calculated as the ratio of the scratch gap at the given time point and the original gap at 0 h. Data are expressed as mean ± SD. * *p* < 0.05, ** *p* < 0.01, *** *p* < 0.001, vs. scrambled controls. NT = Not treated, SCR = scrambled cells, siEphA2 = EphA2-silenced cells.

To functionally validate the role of EphA2 in cell proliferation and migration, we performed silencing experiments in four osteosarcoma cell lines (HOS, KHOS/NP, MNNG/HOS and U-2 OS). We obtained an effective silencing of the protein (Figure 4A,B) and assessed cell viability 72 h after transfection. In all EphA2-silenced osteosarcoma cell lines, we detected a significant reduction in cell viability compared to scrambled controls (*p* < 0.05 in HOS and KHOS/NP, *p* < 0.01 in U-2 OS and *p* < 0.001 in MNNG-HOS, Figure 4C).

Cell migration was investigated by wound healing assay on EphA2-silenced cells compared to their parental counterpart. After 24 and 48 h of transfection, we demonstrated that down-modulation of EphA2 significantly reduced cell migration in all transfected osteosarcoma cell lines, compared to scrambled controls (*p* < 0.05 in HOS and MNNG-HOS, *p* < 0.01 in U-2 OS and *p* < 0.001 in KHOS/NP, Figure 4D,E).

### 2.5. Modulation of MEK6 Impinges on the Activity of Pazopanib and Trametinib Combination

**Figure 5:**
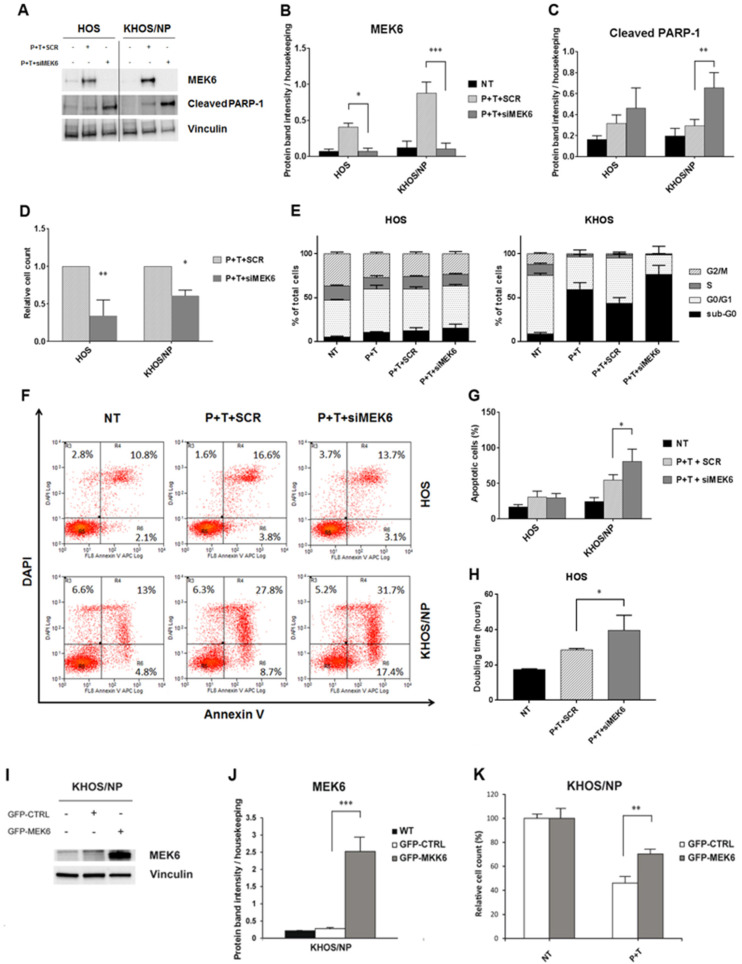
Modulation of MEK6 influenced the anti-proliferative and pro-apoptotic effects of pazopanib + trametinib combination. (**A**), Western blot analysis of MEK6 and cleaved PARP-1 protein expression in osteosarcoma cell lines after 24 h-transfection with specific siRNA against MEK6 and treatment with pazopanib (5 µM) + trametinib (12.5 nM). Vinculin expression was evaluated as loading control. (**B**,**C**), Densitometric analysis of protein band intensity of MEK6 (**B**) and cleaved PARP-1 (**C**) normalized to housekeeping bands. (**D**), Relative cell count measured after 72 h of transfection with specific siRNA against MEK6 or the control siRNA, and calculated as the ratio of transfected or scrambled cells to not treated cells. (**E**), Percentage quantification of FACS analysis of DNA content (cell cycle distribution) in osteosarcoma cells after MEK6-silencing followed by 72 h of treatment with pazopanib (5 µM) and trametinib (12.5 nM). (**F**,**G**), Representative dot plots of cell distribution in early and late phases of apoptosis (**F**) and percentage quantification of annexin V-DAPI staining (**G**) in osteosarcoma cells after MEK6-silencing and 72 h of treatment with the combination of pazopanib (5 µM) and trametinib (12.5 nM). (**H**), Population doubling time of HOS calculated using online software (http://www.doubling-time.com) after MEK6-silencing and 72 h-treatment with the combination of pazopanib (5 µM) and trametinib (12.5 nM). Data are expressed as mean ± SD. * *p* < 0.05, ** *p* < 0.01, *** *p* < 0.001, vs. scrambled controls. NT = Not treated, P + T+SCR = scrambled cells treated with pazopanib (5 µM) + trametinib (12.5 nM), P + T + siMEK6 = MEK6-silenced cells treated with pazopanib (5 µM) and trametinib (12.5 nM) combination. (**I**), Western blot analysis of MEK6 in osteosarcoma cell lines after 24 h of transfection with an ORF GFP-tagged MEK6 expression vector or with control GFP expression vector. Vinculin expression was evaluated as loading control. (**J**), Densitometric analysis of protein band intensity of MEK6 normalized to housekeeping bands. (**K**), Relative cell count measured after 48 h of transfection with ORF GFP-tagged MEK6 expression vector or control GFP expression vector, calculated as the ratio of P + T treated transfected or scrambled cells to not treated cells.

Given MEK6 upregulation by pazopanib + trametinib combination, we validated its functional role by silencing and overexpression experiments in treated osteosarcoma cell lines. As a hallmark of apoptosis, we searched for cleaved PARP-1 after MEK6 silencing in two osteosarcoma cell lines (HOS and KHOS/NP) treated with pazopanib + trametinib combination. After 24 h of drug treatment, MEK6 silencing significantly increased the cleavage of PARP-1, compared to scrambled cells (Figure 5A–C). Similarly, after 72 h of treatment the relative cell counts were significantly lower in MEK6-silenced cells (*p* < 0.01 in HOS and *p* < 0.05 in KHOS/NP, Figure 5D).

These results were also confirmed by flow cytometry analysis. In detail, the down-modulation of MEK6 in KHOS/NP significantly potentiated the proapoptotic effect of the combination (*p* < 0.05, Figure 5E–G), whereas in HOS, pazopanib+ trametinib reduced the percentage of proliferating cells (phase G_2_/M) compared to controls (Figure 5E–G), leading to a significant increase in population doubling-time (*p* < 0.05, Figure 5H). We also explored the impact of MEK6 overexpression. Interestingly, the efficient overexpression of MEK6 (Figure 5I,J) significantly reduced the effect of pazopanib + trametinib combination against KHOS/NP cells when compared to mock controls (*p* < 0.01, Figure 5K).

### 2.6. Pazopanib and Trametinib Mechanism of Action and Correlation with Cell Cycle Control

**Figure 6:**
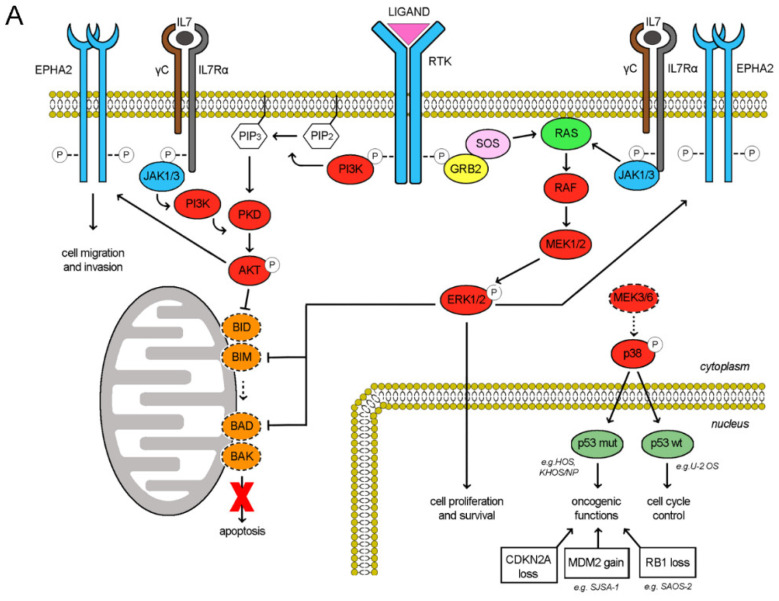

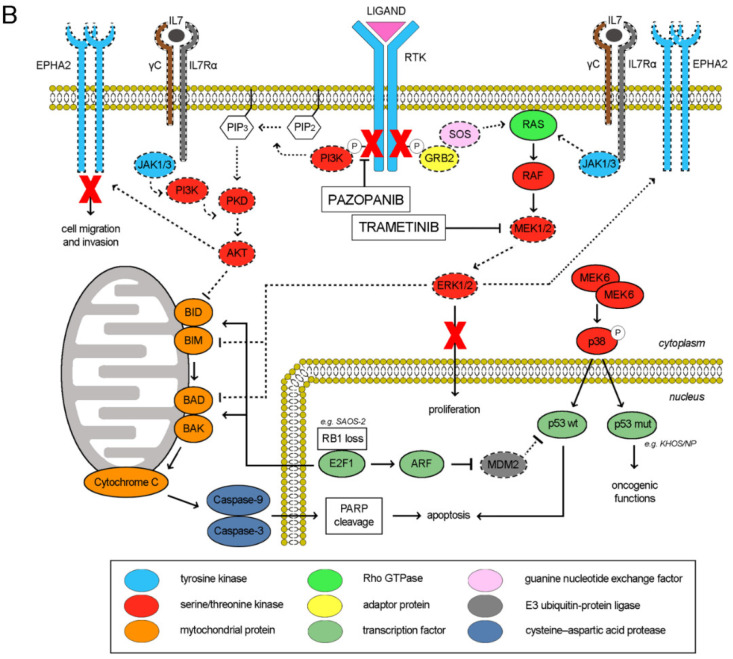
Schematic representation of molecular pathways involved in response to pazopanib and trametinib treatment (**A**), Activated pathways in osteosarcoma cell lines (**B**), Modulation of pathways upon pazopanib and trametinib treatment.

The RTK signaling cascade in osteosarcoma cells could be schematically recapitulated as depicted in Figure 6A. Upon ligand binding, the RTK activation triggers both PI3K/Akt and Ras/ERK signaling cascades, eventually maintaining cell proliferation, survival and invasion. Of note, also the activities of RTK EphA2 and cytokine receptor IL7R are mediated by these two downstreaming pathways [25,26,27,28,29]. In our models, RTK pazopanib targets were differentially expressed and consistently, their downstream pathways were distinctively activated among the different cell lines (Figure 1 and Table S1). We found a direct correlation between RTK pazopanib target expression and sensitivity to pazopanib treatment (Pearson’s score 0.82) as well as between basal ERK activation and sensitivity to trametinib as single agents (Pearson’s score 0.63). On the contrary, no correlation was found between RTK expression, basal ERK/AKT activation and sensitivity to the combination (Table S1). In fact, the pazopanib and trametinib combination suppressed both the PI3K/Akt and the Ras/ERK pathways in additive manner, contemporary inhibiting RTK auto-phosphorylation and blocking MEK1/2 downstream signalling (Figure 6B). Moreover, this drug combination reduced the expression of IL7R and EphA2, while increased MEK6 expression without modifying expression and phosphorylation. EphA2 down-modulation led to inhibited cell migration, while MEK6-upregulation might be a counterbalancing mechanism (Figure 4). Indeed, at basal level (without drugs) osteosarcoma cells expressed a low amount of both MEK6 and its downstream transducer p38α (phosphorylated form). As reviewed by Stramucci et al. [30], this pathway controls p53 activation and in presence of stress stimuli might induce apoptosis in cells with wild type p53 (as in U-2 OS) or activate oncogenic functions in presence of oncogenic mutant p53 (as in HOS, KHOS/NP, MNNG/HOS, Table S2). Of note, all the seven analyzed osteosarcoma cell lines displayed loss of function alteration in the CDKN2A or in RB1 tumor suppressor (Table S2), loss of function or oncogenic mutation of TP53 gene, leading finally to the deregulation of p53 activity and cell cycle control. Different degree of abnormalities in the CDKN2A/RB1/E2F1/ARF/MDM2/p53 signalling could be inferred among the seven tested cell lines and correlated with their response to the drug combination (induction of apoptosis/inhibition of proliferation). Namely, HOS and its derivates KHOS/NP and MNNG/HOS displayed oncogenic mutant p53 and homozygous deletion of N-term CDKN2A (c.del1_449). The increment in MEK6 expression (higher in KHOS/NP and MNNG-HOS; lower in HOS, Table S2 and Figure 3E) upon pazopanib + trametinib treatment could lead to a counterbalancing autohinibitory effect [31] that potentially turned off oncogenic mutant p53 (higher effect in KHOS/NP and MNNG-HOS; lower in HOS) [32]. In MG63, the increment of MEK6 upon drug treatment was modest, but the loss of wild-type p53 functions might explain the absence of expected apoptosis activation. In SAOS-2, the increment of MEK6 was higher but p53 was lost, as RB1 (a well-known feature of this cell line as depicted in Table S2). Therefore, RB1 loss might explain the observed activation of apoptosis that is mediated by E2F1 induced transcription of mitochondrial outer membrane pro-apoptotic proteins (e.g., Bid, Bim, Bad, Bak) [33]. In SJSA-1 cells, wildtype p53 is turned off by MDM2 amplification. Finally, U-2 OS cells displayed an intermediate/low level of MEK6 up-regulation upon treatment and consistently, being p53 wild type, apoptosis was induced (Table S2).

## 3. Discussion

In this work, we demonstrated the potential of pazopanib and trametinib combination as a therapeutic strategy in preclinical models of osteosarcoma both in vitro and in vivo. In this setting, we showed the synergism of this combination, shedding light on the molecular mechanisms behind its antiproliferative and proapoptotic effects. We identified EphA2 and IL-7R as key genes involved in the combination activity. Finally, MEK6 upregulation emerged as a counterbalance by its promotion of resistance to the combination.

Advanced unresectable osteosarcoma is still a fatal disease in children and adults [34]. Even though a complex karyotype and the absence of a clear oncogene addiction [1,35,36,37] made osteosarcomas hard to tackle with target therapies, after our previous report of sorafenib activity in advanced osteosarcoma [38,39], several other TKIs have been tested and have shown intriguing, but short-lived activity [4,5,6,7,8,9]. Moreover, another level of complexity is represented by microenvironment contributions that fuel resistance/rescue pathways [40,41]. The limited activity of further-line chemotherapy, and immunotherapy so far [42], highlighted a rationale for continued pursuit of TKI potentials through exploration of combination therapies to overcome primary or acquired resistance mechanisms. The effective inhibition of activated RTK downstream pathways remains challenging due to signal redundancy. From this perspective, one of the most attractive strategies is the vertical inhibition of RTK pathways known to be activated in advanced osteosarcoma. Our previous results testing the addition of mTOR inhibitors had confirmed the merit of this approach despite a limited overall benefit at the price of a non-negligible toxicity [13,14]. Therefore, we explored the concomitant inhibition of RTKs and the downstream RAS-MAPK pathway with pazopanib and trametinib. In the absence of a direct comparison between the different RTKs in osteosarcoma, our choice was based primarily on the key multitarget specificity and on the more favorable toxicity profile of pazopanib compared with other TKIs that frequently required significant dose reduction when combined with other drugs. Pazopanib alone showed potent anti-angiogenic properties and displayed antiproliferative activity against both endothelial cells and tumor cells expressing its target receptors [15,43,44,45]. Moreover, the combination of pazopanib with the MEK inhibitor trametinib was tested in phase I clinical trials showing non severe adverse events, good tolerability and encouraging preliminary signs of activity in advanced tumors [21,22,23,24].

Our data showed that in the osteosarcoma setting, pazopanib as single agents is differentially active against the seven osteosarcoma cell lines tested and its activity was directly related to RTK expression level on cell surface; while trametinib as single agents was more effective in cell lines displaying activated ERK pathway, as previously shown by Baranski et al. [46]. The combination of pazopanib and trametinib displayed synergistic antitumor effect actively impinging on tumor cell viability through both cell cycle arrest and induction apoptosis. These two drugs targeting directly and indirectly MEK/ERK and PI3K/Akt pathways activated synergistically the pro-apoptotic Bcl-2 family members, particularly Bim a key determinant of apoptosis in response to anticancer targeted agents [47] leading to mitochondrial outer membrane permeabilization and cytochrome c release [48]. Moreover, we enforced these data showing the antitumor activity in vivo against two osteosarcoma xenograft models in NOD/SCID mice and we delved into the molecular mechanisms of the combination. Thus, we screened transcriptional modifications in the expression of both PI3K/Akt and MAPK pathway-related genes. Given our focus, we took advantage of the specificity and sensitivity afforded by the Nanostring technology platform. We monitored the expression of 380 genes, and observed that pazopanib + trametinib combination significantly reduced EphA2 and IL7R and upregulated MEK6 expression. The robustness of these data was validated at protein level. As for EphA2, and in agreement with Fritsche-Guenther data [29], we confirmed the role of this protein in proliferation and migration of osteosarcoma cells using silencing experiments. For the first time, we also showed that the activity of pazopanib + trametinib is related to the inhibition of this oncogene. The other gene found to be related to the combination response was IL-7R. This receptor has been described as a driver gene in T-cell acute lymphoblastic leukemia promoting cell transformation and tumor formation [27,49,50], whereas its role in solid tumors is less well-defined. In osteosarcoma cell lines, its expression was initially demonstrated several years ago [51], but its functions had yet to be investigated. Our data suggest its involvement in TKI activity and prompt further studies on its role as a potential target in osteosarcoma. This asset might be involved in tumor-stroma crosstalk [40,41] and important implication might be obtained by pazopanib–trametinib treatment on tumor immune microenvironment even modulating tumor response in vivo. Giving the characteristics of our in vivo models (obtained by engraftment of human osteosarcoma cell lines in immunodeficient NOD/SCID mice) we cannot deepen in this potentially relevant aspect within this work. Further studies with syngeneic models and the molecular analysis of tumor specimen derived from treated patients might help in unveiling the different TKI effects on tumor microenvironment depending on the entire biological context.

Finally, our results highlight an upregulation of MEK6 in response to pazopanib + trametinib. We validated this original finding by silencing and overexpression, confirming MEK6 association with drug resistance. In particular, our data suggest that MEK6 upregulation might have an escape role in antiproliferative and proapoptotic effects of pazopanib and trametinib treatment. This observation is consistent with that described in gastrointestinal cancers (esophageal, stomach, and colon), and in breast cancer, supporting a direct role in tumorigenesis [30,52,53]. On the other hand, inactivation of MEK6 in ovarian cancer may induce metastatic spread [54]. MEK6 is involved in cell cycle control, proliferation, senescence and apoptosis mainly through p38-MAPK [55]. The interplay between MEK6 and p38 might be different according to tumor type and it eventually remains to be fully elucidated. Since we did not observe any change in p38α expression and phosphorylation in osteosarcoma cell lines treated with pazopanib + trametinib, we could infer that MEK6 may influence cell response to the drug combination by modulating other downstream targets, such as other p38 isoforms. As reviewed in Stramucci et al. [30], the MEK6-p38 pathway controls p53 activation and in presence of stress stimuli might induce apoptosis in cells with wild type p53 or activate oncogenic functions in presence of mutant p53. Of note, all seven of the osteosarcoma cell lines displayed deregulated CDKN2A/RB1/E2F1/p14ARF/MDM2/p53 axis, upsetting cell cycle control and apoptosis. Different degrees of abnormalities in the CDKN2A/MDM2/p53/RB1/E2F1 signalling may correlate with the peculiar response to the drug combination that we observed in the seven osteosarcoma cell lines (induction of apoptosis/inhibition of proliferation). In particular E2F1 in the absence of in RB1-defective tumors might lead to transcription of pro-apoptotic Bcl-2 family members [33], while other defects along the pathway even in presence of wild type RB1 might retain anti-apoptotic proteins [56] In view of these considerations, MEK6 could represent a druggable target worthy of exploration and capable of widening the vertical inhibition of RTK pathways. Importantly, the characterization of molecular context might be crucial to infer clinical activity of this strategy. In particular, the deregulation of p53 and RB1 axis, a frequent event in osteosarcoma [1,57,58], might define the crossroad of intersecting pathways shaping the ultimate cellular fate during drug treatment [30,33,59]. Further studies with MEK6 specific inhibitors in combination with TKIs are warranted. Nonetheless, even in presence of MEK6 upregulation, pazopanib and trametinib combination is still effective thanks to the multiple inhibition of RTK pathways both upstream at RTK level (among which EphA2 and IL7R) and downstream at PI3K/Akt and MEK/ERK levels.

## 4. Materials and Methods

### 4.1. Cell Cultures and Drugs

Seven human osteosarcoma cell lines (HOS, KHOS/NP, MG63, MNNG/HOS, SAOS-2, SJSA-1, and U-2 OS) were purchased from the American Type Culture Collection (ATCC, Manassas, VA, USA) and cultured in the following media: MNNG/HOS and KHOS/NP in Eagle’s Minimum Essential Medium (EMEM, Sigma-Aldrich, Merck KGaA, Darmstadt, Germany), SJSA-1 in RPMI-1640 Medium (Sigma-Aldrich), and HOS, MG63, SAOS-2, and U-2 OS in Dulbecco’s Modified Eagle Medium (DMEM) with 4500 mg/L glucose (Sigma-Aldrich). All media were supplemented with 100 U/mL penicillin, 100 μg/mL streptomycin, and 10% heat inactivated fetal bovine calf serum. All were cultured at 37 °C in a humidified chamber containing 5% CO_2_; their identity was confirmed using the StemElite ID system (Promega Corporation, Madison, WI, USA) according to the manufacturer’s instructions and the ATCC STR profile database.

For in vitro experiments, pazopanib and trametinib (Selleckchem, Munich, Germany) stock solutions were dissolved in dimethylsulfoxide (DMSO, Sigma-Aldrich), stored at −80 °C, and diluted in fresh media immediately before use. For in vivo experiments, both drugs were purchased from Carbosynth (Compton, UK). The pazopanib solution was prepared by resuspending the powder in 0.5% hydroxypropylmethylcellulose (Sigma-Aldrich) with 1% Tween-80 (Sigma-Aldrich), the pH was adjusted to 1.3–1.5, and the solution was sonicated for 5 min before oral gavage. Trametinib was dissolved in DMSO and then diluted in water immediately before administration.

### 4.2. Flow Cytometry Analysis

RTKs expression in osteosarcoma cell lines was evaluated using the following conjugated antibodies: anti-human KIT/CD117–PE (DAKO, Agilent, Santa Clara, CA, USA), anti-human FGFR3-PE, anti-human PDGFRβ-APC, anti-human FGFR2–APC, anti-human PDGFRα-PE, anti-human VEGFR3-APC, anti-human VEGFR1–PE, and anti-human KDR/VEGFR2–APC (R&D Systems, Minneapolis, MN, USA). Isotype controls (Ig isotype control cocktail, BD Pharmingen, Franklin Lake, NJ, USA) were used to set the negative gate for cells (Mean Fluorescence Intensity ≤ 10).

For cell cycle analysis, osteosarcoma cells were plated in complete culture medium and treated with 10 µmol/L pazopanib, 25 nmol/L trametinib, and their combination. After 72 h of treatment, the effect of the drug treatment on the cell cycle was determined by DNA content evaluation after propidium iodide staining. Drug-treated cells were harvested by trypsin–EDTA, washed in PBS, and fixed for 24 h in 70% ethanol at −20 °C. Next, the cells were incubated with propidium iodide staining solution (10 µg/mL), 100 µg/mL RNase A, and 0.1% (*v*/*v*) Triton X-100 (Sigma-Aldrich) in PBS for 30 min at room temperature.

Apoptosis in the osteosarcoma cell lines was evaluated using Annexin V/propidium iodide (PI) (or 4’,6-Diamidine-2’-phenylindole dihydrochloride, DAPI) after 72 h of treatment. Briefly, cells were detached by trypsin–EDTA, washed in PBS, and incubated in cold binding buffer (150 mmol/L NaCl_2_, 10 mmol/L CaCl_2_ and 10 mmol/L HEPES) for 30 min. Next, 2 × 10^5^ cells were incubated with 2.5 µL of APC-labeled Annexin V (eBioscence, Thermo Fisher Scientific, Carlsbad, CA, USA) and PI (0.5 mg/mL, Sigma-Aldrich) or DAPI (2 µg/mL, Thermo Fisher Scientific) for 1 h at 4 °C. All samples were processed by a Cyan ADP flow cytometer (Beckman Coulter, Brea, CA, USA) and analyzed using Summit v4.3 software (Beckman Coulter).

### 4.3. Western Blot

To obtain total protein extracts, cells were detached with trypsin-EDTA in PBS (EuroClone, Pero, MI, Italy), washed with cold PBS, and solubilized at 1 × 10^7^ cells/mL in Lysis Buffer 6 (R&D Systems, Minneapolis, MN, USA). Cells were mechanically lysed on ice with a pipette and rocked gently for 30 min at 4 °C and then clarified to remove cell debris. Protein concentration was determined using a bicinchoninic acid assay (BCA Protein Assay, Thermo Fisher Scientific); absorbance levels were measured using BioPhotometer (Eppendorf, Hamburg, Germany). Protein samples of 10 to 30 µg were resolved by electrophoresis on 4–15% mini-PROTEAN^®^TGX™ PreCast gels (Bio-Rad Laboratories, Hercules, CA, USA), and then transferred to a nitrocellulose membrane via the Trans-Blot^®^ Turbo™ Transfer System (Bio-Rad Laboratories). Non-specific sites were blocked with BSA 10% for 1 h at room temperature after which the membranes were incubated overnight at 4 °C with the following primary antibodies: anti-human MEK6, anti-human EphA2, anti-human P-Akt (Ser473), anti-human P-ERK 1/2 (Thr202/Tyr204), anti-human ERK 1/2, anti-human Akt, anti-human Bid, anti-human caspase 3, anti-human Bim, anti-human Bik, anti-human Bad, anti-human BaK, anti-human cleaved caspase 3, anti-human cleaved PARP, anti-human p38, anti-human P-p38 (Thr180/Tyr182), anti-human β-Actin (Cell Signaling Technology, Leiden, The Netherlands), anti-human CD127 (IL-7Rα, Invitrogen by Thermo Fisher Scientific), and anti-human vinculin (Sigma-Aldrich). Specific signals were visualized with HRP–conjugated secondary antibodies (Jackson Immuno Research Laboratories, West Grove, PA, USA) and detected using the Bio-Rad Chemidoc™ Touch Imaging System (Bio-Rad Laboratories) following exposure to Clarity™ Western ECL substrate (Bio-Rad Laboratories). Band intensity was quantified by ImageLab v5.2.1 (Bio-Rad Laboratories), using vinculin and β-actin as normalizers.

### 4.4. Colony Formation and Cell Viability Assays

To assess the antitumor effects of pazopanib and trametinib against osteosarcoma cell lines, single cell-derived colonies were obtained by plating 500 cells per well on 12-well plates in complete medium. After 24 h, cells were treated with pazopanib (2.5–10 µmol/L) and trametinib (6.125–25 nmol/L) as single agents and as a constant combination. After 7 days, colonies were stained with 0.1% crystal violet (Sigma-Aldrich) and their surface areas were quantified using Image J software.

For viability assays, osteosarcoma cell lines were seeded in 96-well plates (1500 cells/well). After 24 h, scalar dilutions of pazopanib (20–0.625 µmol/L), trametinib (from 50 to 6.25 nmol/L), and their combination were administered. Cell viability was evaluated after 72 h with the Cell Titer-Glo^®^ luminescent cell viability kit (Promega). The measurement of ATP content in viable cells was proportional to the number of viable cells, and detectable by luciferin-luciferase reaction. The luminescence signal was detected by Synergy HT luminometer (Biotek Instruments, Winooski, VT, USA) and analyzed by Gen5 v1.09 software (Biotek Instruments). Post-treatment viable cell proportions were obtained after normalization with the untreated controls and the concentration inhibiting 50% of the cell population (IC50) was calculated using CalcuSyn Software (Biosoft, Cambridge, UK). Drug synergism was evaluated by the Chou-Talalay method.

### 4.5. Cell Transfection and Transduction

HOS, KHOS/NP, MNNG/HOS, and U-2 OS cells were grown in 6-well plates to 50% confluence, after which the medium was changed and cells were transfected in Opti-MEM^®^I Reduced Serum Medium (Gibco by Life Technologies, Paisley, UK) without antibiotics. According to the manufacturer’s instructions, 30 pmol of 27mer siRNA duplexes specific for MEK6 (SR303765, Gibco Technologies, Rockville, MD, USA) or EPHA2 (SR301367, OriGene, Rockville, MD, USA) or Control siRNA (Universal scrambled negative control siRNA duplex, OriGene) were mixed with an appropriate amount of Lipofectamine^®^RNAiMAX (Thermo Fisher Scientific) diluted in Opti-MEM^®^I Reduced Serum Medium. After 10 min of incubation at room temperature, the transfection mixture was added dropwise to the cells to a final concentration of 10 nM. After 6 h, the transfection medium was removed and cells were cultured in the presence/absence of the studied drugs for 24–48–72 h. Next, cells were then detached with trypsin-EDTA and counted using TC10™ Automated Cell Counter (Bio-Rad), excluding the dead cells with Trypan Blue Stain (0.4%, NanoEntek, Waltham, MA, USA). Non-transfected cells and cells transfected with the negative control siRNA (scrambled) were used as controls. Silencing efficacy was checked by western blot, by measuring protein expression of MEK6 and EphA2 24 h post-transfection. To assess the EphA2 and MEK6 silencing effect on cell growth, osteosarcoma cell lines were transfected with EphA2 siRNA or MEK6 siRNA in 96-well plates (described above), and cell viability was evaluated 72 h after transfection using the Cell Titer-Glo^®^ luminescent cell viability kit. Non-transfected cells and those transfected with negative control siRNA (scrambled) served as controls.

KHOS/NP cells were plated in 6-well plates at 5.0 × 10^4^ cells/well and in vitro stably transfected with MEK6 or GFP (Lenti ORF clone of human MEK6 mGFP tagged, RC204481L4, OriGene) overexpressing lentiviral particles (180 ng/mL of p24 in 2 mL of total medium) in the presence of polybrene infection reagent (8 µg/mL, Sigma Aldrich Darmstadt, Germany) diluted in the culture medium. Lentivirus Titer (p24) was tested with the HIV-I p24 ELISA 96-well Kit (Alliance^®^, cat.NEK050A001KT). After 12 h, transfection medium was removed, cells were kept growing for 48 h, then lysed with Lysis buffer 6 (R&D Systems) and overexpressed MEK6 and control GFP proteins were checked by western blotting. The effect of pazopanib (1.25 µM) and trametinib (3.125 nM) combination in MEK6 overexpressing cells was evaluated by cell proliferation assays. Transduced KHOS/NP cells (8.5 × 10^4^ cells) were plated in 6-well plates; and then treated with pazopanib (1.25 µM) and trametinib (3.125 nM) as single agents and in combination for 48 h. Cell viability was evaluated by cell counting (trypan blue dye exclusion) and normalized to each untreated controls.

### 4.6. Wound Healing Assay

To evaluate EphA2 involvement in cell migration, confluent monolayers of silenced EphA2- cell lines were scratch-wounded with a 200 µL micropipette tip. Dislodged cells and debris were removed by washing cells with medium. Images of wound closure were taken immediately after scratching and at 24 and 48 h post-wounding using an Axio Vert.A1-Inverted Microscope (Carl Zeiss, Aalen, Germany), equipped with the True Chrome HD II camera (Tiesselab, Milano, Italy). Cell migration was measured at relative scratch gaps by ImageJ software, calculating the ratio of the scratch gap at the given point in time and the original gap at 0 h.

### 4.7. RNA Extraction and Nanostring^®^ nCounter Assay

Three osteosarcoma cell lines (HOS, MNNG/HOS, and U-2 OS) were treated for 24 h with pazopanib 10 µM and trametinib 25 nM alone or in combination, and then were subjected to RNA extraction, using the Maxwell^®^ RSC miRNA Tissue Kit AS1460 (Promega). Namely, 10^6^ cells were lysed with 1-Thioglycerol/Homogenization Solution. RNA samples were prepared following the manufacturer’s instructions of Maxwell^®^ RSC Instrument. RNA purity (DeNovix DS-11+ Spectrophotometer, DeNovix Inc., Wilmington, DE, USA), concentration (Qubit^®^3.0 Fluorometer (Invitrogen by Life Technologies, Eugene, OR, USA), and fragmentation (Agilent 2100 bioanalyzer, Agilent Technologies, Wilmington, DE, USA) were determined.

Gene expression modulation by drug administration was determined by NanoString^®^ nCounter Technology (NanoString Technologies, Seattle, WA, USA) using the nCounter^®^Vantage 3D™RNA MAPK-PI3K Pathways Panel. As per the manufacturer’s protocol, samples were prepared for hybridization with Prep Station processing, nCounter^®^ Analysis System counting, and nSolver software, v3.0 analysis. Transcript copies were normalized using the geometric mean of 12 housekeeping genes for reference; the log2-fold changes of gene expression were calculated comparing treated samples with untreated controls.

### 4.8. Mice Xenograft Models

We evaluated antitumor activity of pazopanib, trametinib, and their combination in non-obese diabetic/severe combined immunodeficient (NOD/SCID) mice (Charles River, Wilmington, MA, USA) subcutaneously injected with 10^6^ cells (MNNG/HOS or KHOS) into the right flank at 6 weeks of age. When the tumors became palpable (approximately after three weeks), seven mice per group were randomized to receive by oral gavage: (a) pazopanib (40 mg/kg, 5 days/week), (b) trametinib (0.1 mg/kg, 5 days/week), (c) their combination, and (d) vehicle only (control group). Tumor growth was monitored weekly with a digital caliper. Volumes (V) were calculated according to the following equation: V = (largest diameter × smallest diameter)^2^/2. After 24 days of treatment, the mice were sacrificed by lethal anesthesia (isoflurane + oxygen). All procedures were conducted in accordance with the protocol approved by the Institutional Ethics Review Board (IRB) and by the Italian Ministry of Health (Aut. Min. 178/201S-PR). Animals were maintained in cage microinsulators and handled under sterile conditions at the animal facility of the Institute for Cancer Research and Treatment (Candiolo, Italy).

### 4.9. Statistical Analysis

All in vitro experiments were performed at least three times. Descriptive analyses of osteosarcoma features were reported as mean ± SEM. Means, SDs, and 95% confidence intervals were calculated using GraphPad Prism software (La Jolla, CA, USA). Differences among the treatment groups were analyzed by two-tailed Student’s *t* test, one-way ANOVA, and two-way ANOVA with post-hoc Bonferroni’s correction for multiple tests. The concentration inhibiting 50% of cell growth (IC50) with its 95% confidence intervals (95% CI), and the drug synergism, expressed as combination index (CI) ± estimated SD, were calculated by CalcuSyn software (Biosoft) based on the Chou–Talalay method. Pearson score was calculated to test correlation between RTK expression level (fluorescence intensity) or P-AKT or P-ERK band intensity normalized to total protein and housekeeping and IC50 of pazopanib or trametinib as single agent and in combination.

## 5. Conclusions

In conclusion, the combination of pazopanib and trametinib deserves clinical investigation in the context of advanced osteosarcoma. Our data support further study of EphA2 and IL-7R as predictive biomarkers to improve patient selection. In fact, MEK6 could represent a new druggable target to overcome resistance to this combination and deserve further exploration also in the context of other TKI treatment.

## Figures and Tables

**Table 1:** Cell viability assay on seven osteosarcoma cell lines treated with pazopanib, trametinib, and their constant combination. Concentrations inhibiting 50% of the cell growth (IC50) values with 95% confidence intervals after 72 h of treatment with scalar doses of pazopanib (20, 10, 5, 2.5, and 1.25 µM), trametinib (50, 20, 10, 5, and 2.5 nM), and their constant combination. Drug synergism is expressed as a combination index (CI), calculated at IC50; Est. SD = estimated standard deviation.

Cell Line	IC_50_ Pazopanib (95% CI) µM		IC_50_ Trametinib (95% CI) nM		Combination Index ± Est. SD
	Single Agent	Combination	Single Agent	Combination	
HOS	14.26 (12.43–16.35)	8.99 (8.88–9.10)	>50	22 (22–23)	0.74 ± 0.09
KHOS/NP	16.98 (14.21–20.32)	0.88 (0.42–1.84)	4 (2–6)	2 (1–5)	0.57 ± 0.34
MG63	9.84 (8.64–11.20)	3.36 (2.92–3.86)	45 (28–62)	8 (7–10)	0.53 ± 0.06
MNNG-HOS	10.96 (9.39–12.8)	3.23 (2.25–4.62)	25 (22–28)	8 (6–11)	0.62 ± 0.12
SAOS-2	>20	2.77 (2.20–3.48)	24 (7–74)	7 (5–9)	0.32 ± 0.04
SJSA-1	6.00 (5.45–6.6)	4.53 (4.14–4.96)	18 (15–21)	11 (10–12)	0.99 ± 0.06
U-2OS	10.32 (9.34–11.41)	4.57 (4.25–4.91)	>50	11 (11–12)	0.56 ± 0.04

## Supplementary Materials

The following are available online at https://www.mdpi.com/2072-6694/12/6/1519/s1, Table S1: Summary of receptor tyrosine kinase (RTK) expression level (sum of fluorescence intensity of each tested RTK), P-AKT and P-ERK band intensity, IC_50_ of pazopanib (P), trametinib (T) as single agents and in combination; and their relative Pearson’s correlation. Table S2: Summary of crucial gene alterations, induction of MEK6 upon drug treatment and correlation with cellular outcomes (induction of apoptosis and potential molecular mechanism evoked) in each cell lines. Figure S1: The combination of pazopanib and trametinib induced apoptosis in osteosarcoma cell lines. Figure S2: Pazopanib + trametinib combination induced apoptosis in osteosarcoma cells by modulating the expression and activation of Bcl-2 family pro-apoptotic members and the cleavage of caspase 3 and PARP. Figure S3: Pazopanib + trametinib combination did not significantly change p38 activation.
