# Supplementary material for: Pazopanib and Trametinib as a Synergistic Strategy against Osteosarcoma: Preclinical Activity and Molecular Insights
Source: Cancers (Basel). 2020 Jun 10;12(6):1519. doi: 10.3390/cancers12061519 (PMC7352822; doi:10.3390/cancers12061519)
Supplement: Supplementary file 1 [file cancers-12-01519-s001.zip › cancers-823818-supplement.pdf]

cancers

MDPI

Supplementary Materials: Pazopanib and Trametinib as a Synergistic Strategy against Osteosarcoma: Preclinical Activity and Molecular Insights

Table S1. Summary of receptor tyrosine kinase (RTK) expression level (sum of fluorescence intensity of each tested RTK), P-AKT and P-ERK band intensity, IC50 of pazopanib (P), trametinib (T) as single agents and in combination (above); and their relative Pearson's correlation (below).

Cell Line	RTK Expression Level	P-AKT	P-ERK	IC50 P (µM)	IC50 T (nM)	IC50 P combination (µM)	IC50 T combination (nM)
HOS	260	2.23	1.82	14.26	>50.00	8.99	22.00
KHOS/NP	192	1.42	2.65	16.98	4.00	0.88	2.00
MG63	237	1.21	0.82	9.84	45.00	3.36	8.00
MNNG-HOS	205	1.89	1.73	10.84	25.00	3.23	8.00
SAOS-2	165	0.62	1.24	>20.00	24.00	2.77	7.00
SJSA-1	337	0.41	1.34	6.00	18.00	4.53	11.00
U-2OS	240	1.32	0.56	10.32	>50.00	4.57	11.00

Pearson Correlation		P-AKT vs IC50	P-ERK vs IC50
P	- 0.82	0.11	0.42
T	0.18	0.38	-0.67
P+T	0.49	0.44	-0.19

Table S2. Summary of crucial gene alterations, induction of MEK6 upon drug treatment and correlation with cellular outcomes (induction of apoptosis and potential molecular mechanism evoked) in each cell lines.

Cell Line	KRAS	Induction of Apoptosis	Induction of MEK6	Potential Molecular Outcome upon Treatment	TP53 STATUS	CDKN2A/RB Pathway	References
HOS		<50%	low	oncogenic mutant p53 might be active	c.467G>C p.R156P (COSS907060) gain-of function : oncogenic	Homozygous for CDKN2A deletion c.1_471del471	DOI: 10.1002/GCC.20717
KHOS/NP	overexpressed murine Kirstein sarcoma virus	>50%	high	oncogenic mutant p53 is turn off by MEK6 upregulation	c.467G>C p.R156P (COSS907060) gain-of function: oncogenic	Homozygous for CDKN2A deletion c.1_471del471	DOI: 10.18632/oncotarget.6634
MG63		<50%	low	p53 loss	loss of p53	Homozygous for CDKN2A deletion c.1_471del471	DOI: 10.1128/mcb.10.11.5772
MNNG-HOS		>50%	high	oncogenic mutant p53 is turn off by MEK6 upregulation	c.467G>C p.R156P (COSS907060) gain-of function: oncogenic	Homozygous for CDKN2A deletion c.1_471del471	DOI: 10.1002/GCC.20717
SAOS-2		>50%	high	rb1 loss activate E2F1 (tansduction of apoptotic mytochondrial protein)	c.1_1182del1182 loss- of- function	loss of RB1 (c.2212_2787del576)	DOI: 10.1128/mcb.10.11.5772
SJSA-1	c.183A>T P. Q61H (COSV55498802)	<50%	high	MDM2 amplification ihibited wild type p53	WT (COSMIC 909717) MDM2 amplification	Hemyzygous deletion of CDKN2A	DOI: 10.1126/science.1092472
U-2OS		>50%	intermediate/ low	wild type p53 contributed to apoptosis induction	WT	loss of CDKN2A	DOI: 10.1158/1541-7786.MCR-17-0089

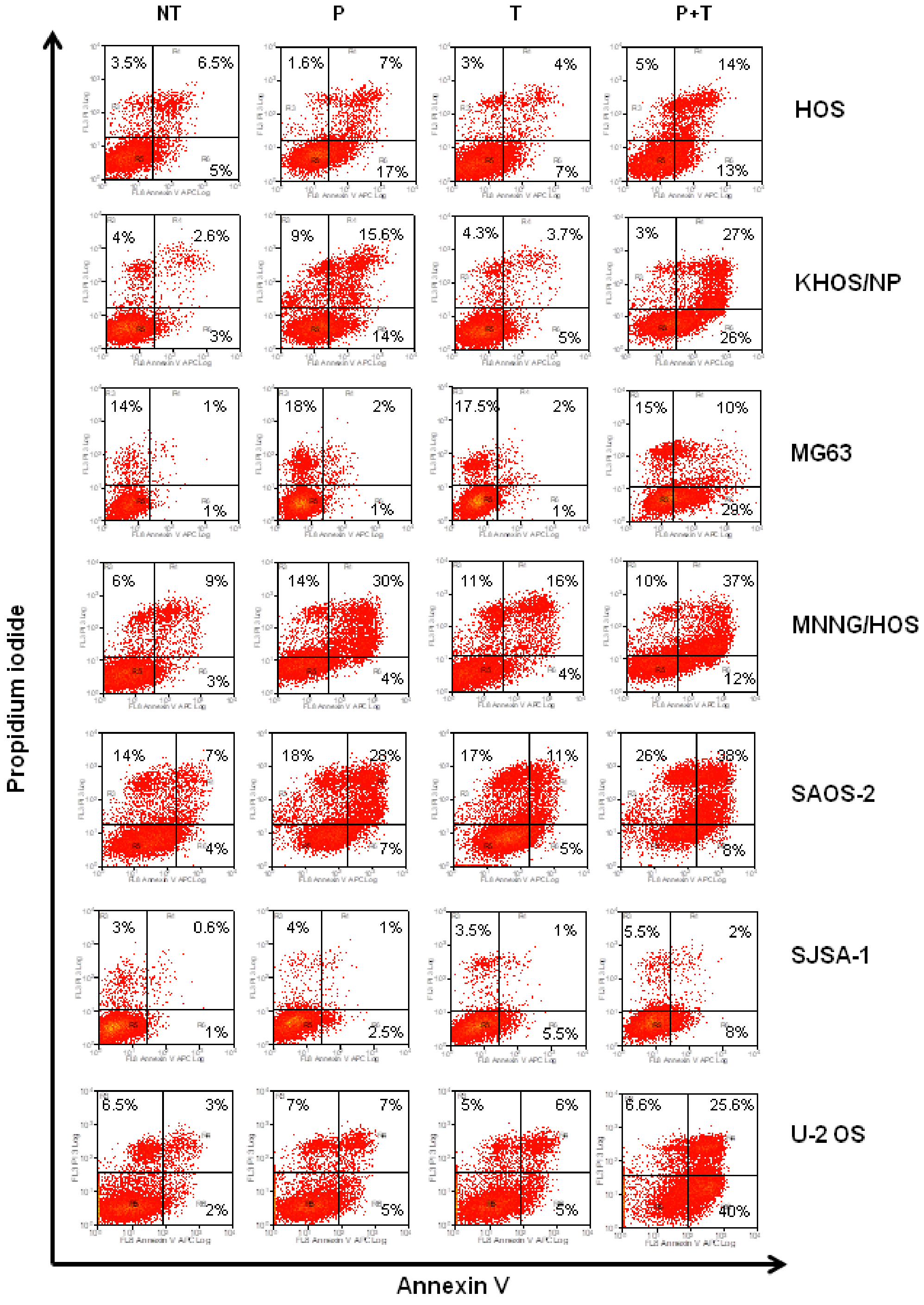

Figure S1. The combination of pazopanib and trametinib induced apoptosis in osteosarcoma cell lines. Representative dot plots of cell distribution in early and late phases of apoptosis after 72 hours of treatment with pazopanib (10µM) and trametinib (25nM) as single agents and in combination. NT = not treated. P = pazopanib. T = trametinib. P+T = pazopanib+trametinib combination.

.

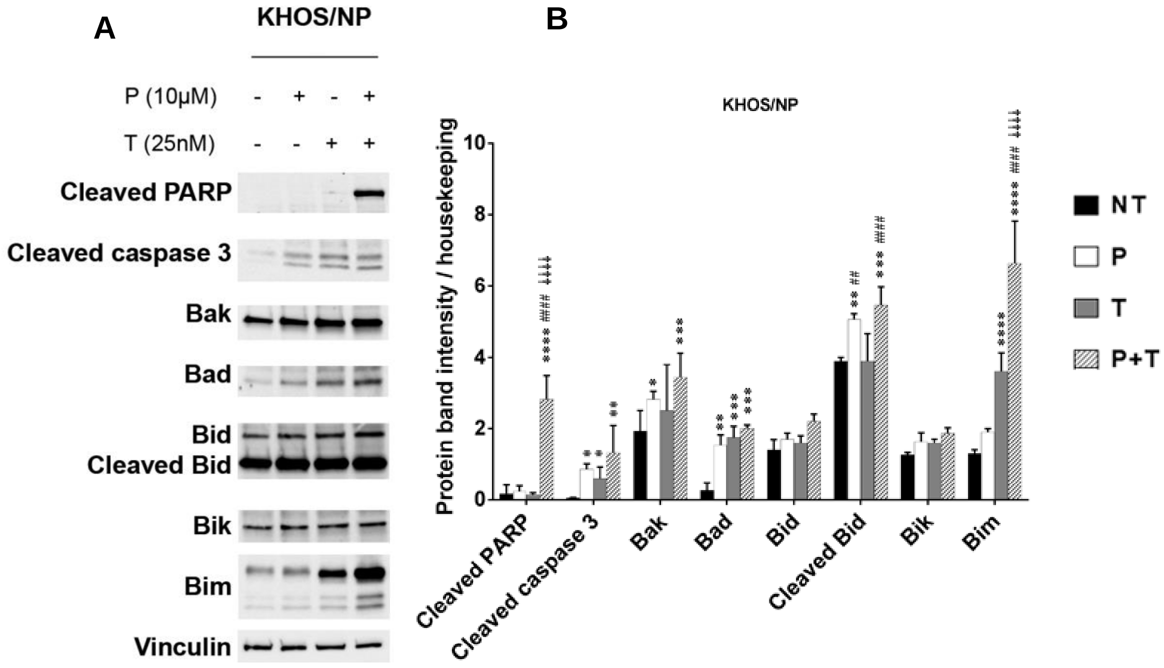

Figure S2. Pazopanib + trametinib combination induced apoptosis in osteosarcoma cells by modulating the expression and activation of Bcl-2 family pro-apoptotic members and the cleavage of caspase 3 and PARP. (**A**) Representative western blot analysis of protein expression/activation/cleavage after 24 hour-exposure to pazopanib (10 μM) and trametinib (25 nM) as single agents and in combination. (**B**) Densitometric analysis of protein expression normalized to housekeeping bands. Data are expressed as mean ± SD. Independent experiments were performed three times in KHOS cells. * $p < 0.05$, ** $p < 0.01$, *** $p < 0.001$, **** $p < 0.0001$ vs. controls; ## $p < 0.01$, #### $p < 0.0001$ vs. trametinib; †††† $p < 0.0001$ vs. pazopanib. NT = not treated, P = pazopanib, T = trametinib, P+T = pazopanib+trametinib combination.

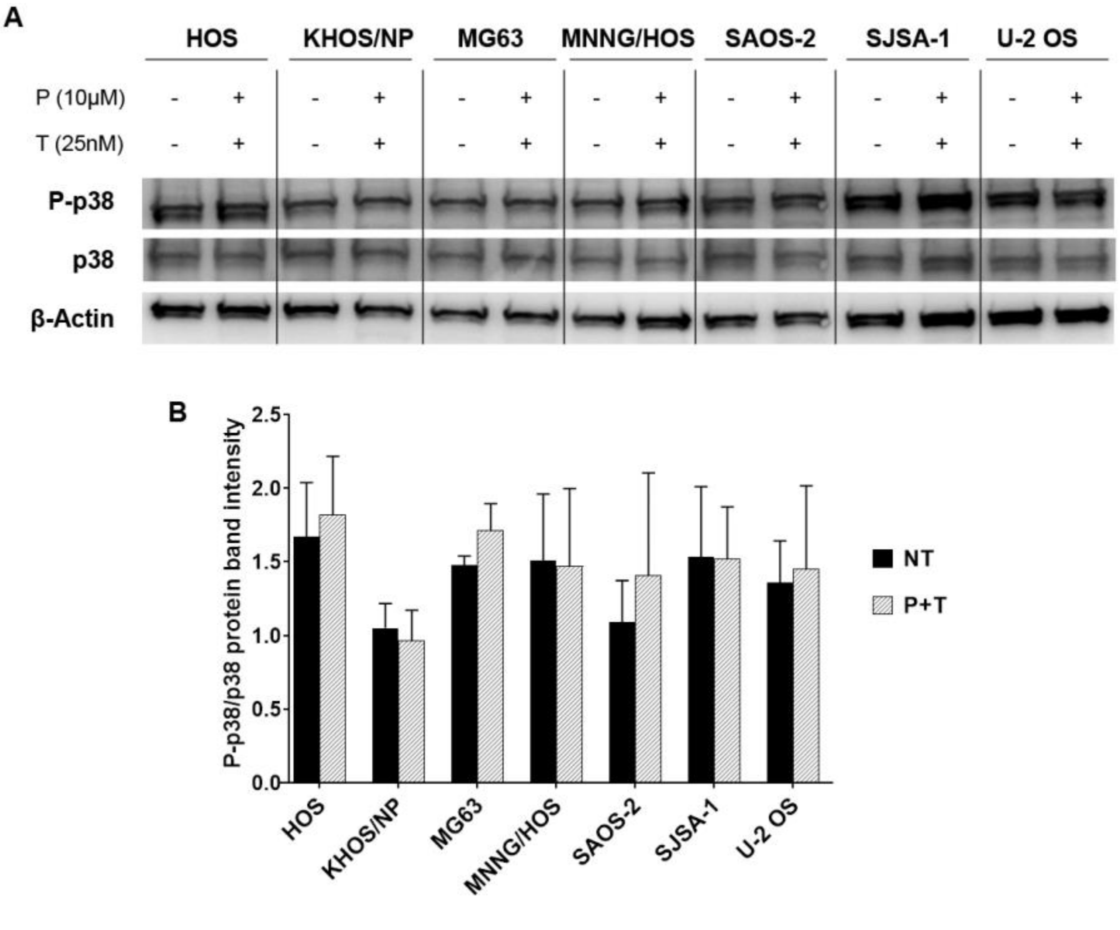

Figure S3. Pazopanib+trametinib combination did not significantly change p38 activation. (**A**) Representative western blot images. (**B**) Densitometric analysis of protein band intensity normalized to housekeeping bands. Data are expressed as mean ± SD of two independent experiments.

© 2020 by the authors. Licensee MDPI, Basel, Switzerland. This article is an open access article distributed under the terms and conditions of the Creative Commons Attribution (CC BY) license (http://creativecommons.org/licenses/by/4.0/).
