# Supplementary material for: Pazopanib and Trametinib as a Synergistic Strategy against Osteosarcoma: Preclinical Activity and Molecular Insights
Source: Cancers (Basel). 2020 Jun 10;12(6):1519. doi: 10.3390/cancers12061519 (PMC7352822; doi:10.3390/cancers12061519)
Supplement: Supplementary file 1 [file cancers-12-01519-s001.zip › cancers-823818-Western blot images.pdf]

Figure 1 B

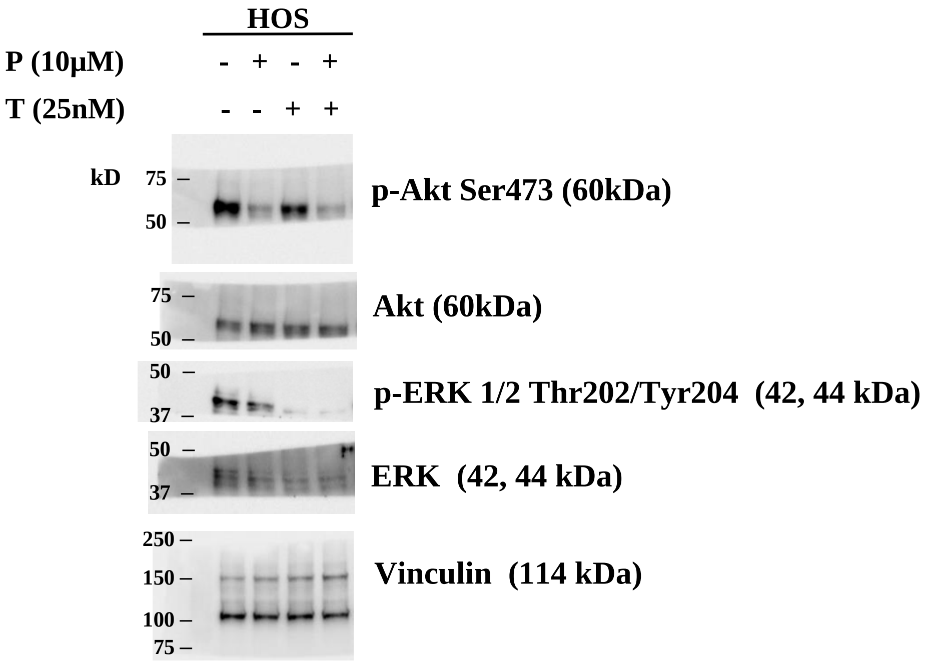
HOS
P (10µM) - + - +
T (25nM) - - + +
kD
75
50
p-Akt Ser473 (60kDa)
75
50
Akt (60kDa)
50
37
p-ERK 1/2 Thr202/Tyr204 (42, 44 kDa)
50
37
ERK (42, 44 kDa)
250
150
100
75
Vinculin (114 kDa)

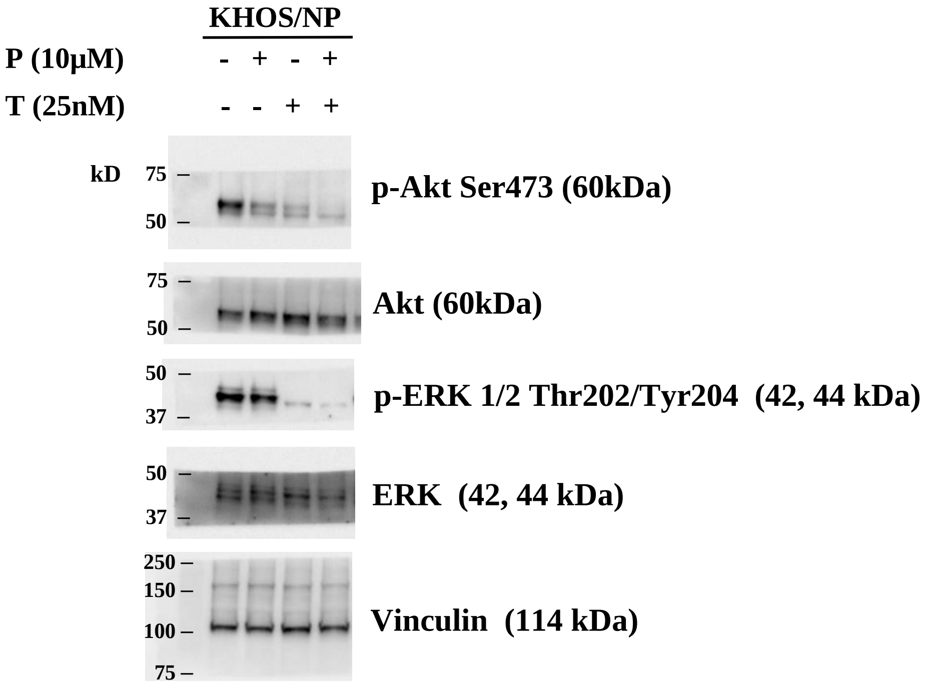
KHOS/NP
P (10µM) - + - +
T (25nM) - - + +
kD
75
50
p-Akt Ser473 (60kDa)
75
50
Akt (60kDa)
50
37
p-ERK 1/2 Thr202/Tyr204 (42, 44 kDa)
50
37
ERK (42, 44 kDa)
250
150
100
75
Vinculin (114 kDa)

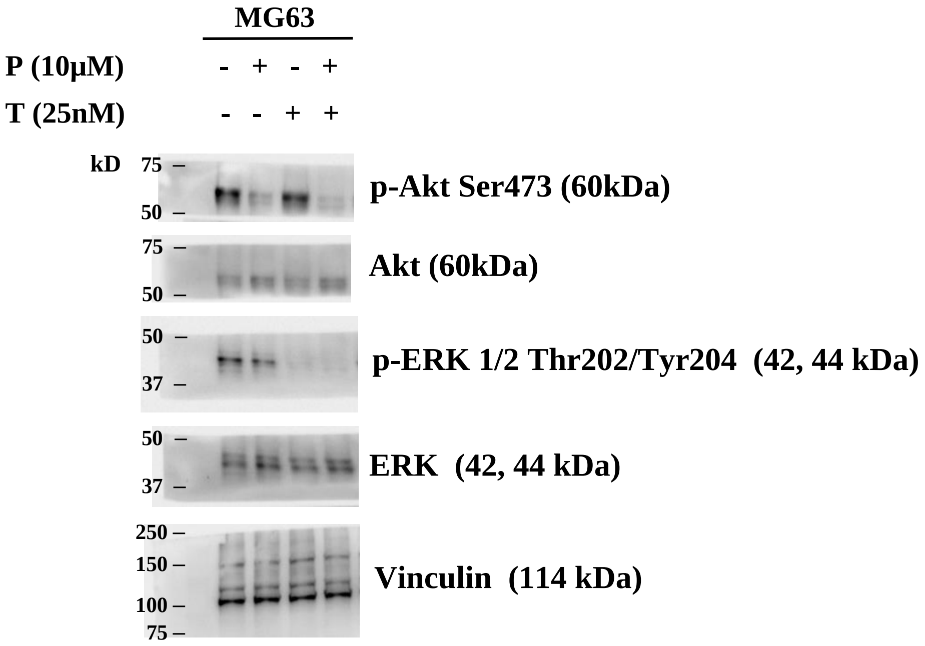
MG63
P (10µM) - + - +
T (25nM) - - + +
kD
75 –
50 –
p-Akt Ser473 (60kDa)
75 –
50 –
Akt (60kDa)
50 –
37 –
p-ERK 1/2 Thr202/Tyr204 (42, 44 kDa)
50 –
37 –
ERK (42, 44 kDa)
250 –
150 –
100 –
75 –
Vinculin (114 kDa)

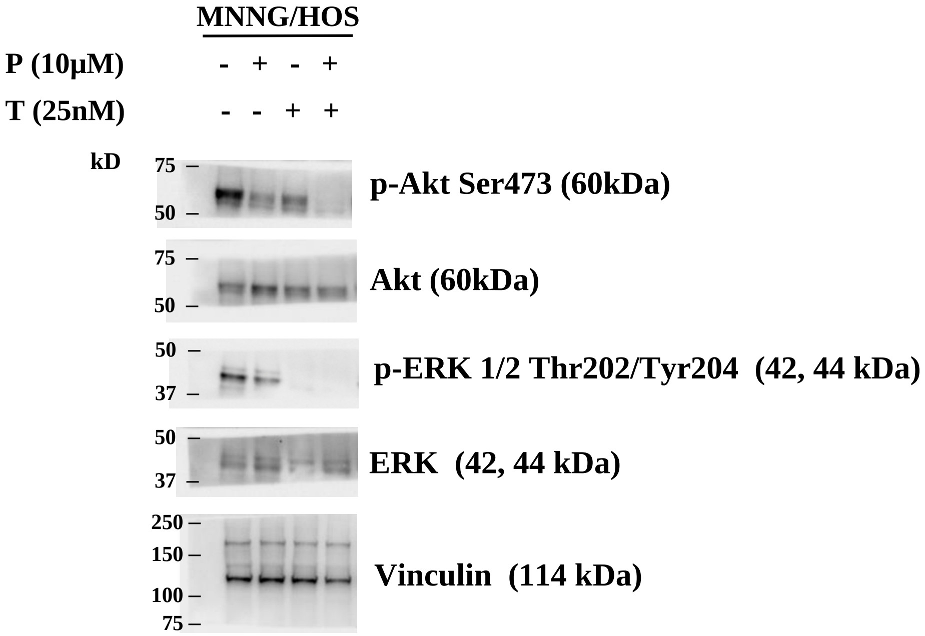
MNNG/HOS
P (10µM) - + - +
T (25nM) - - + +
kD
75 –
50 –
p-Akt Ser473 (60kDa)
75 –
50 –
Akt (60kDa)
50 –
37 –
p-ERK 1/2 Thr202/Tyr204 (42, 44 kDa)
50 –
37 –
ERK (42, 44 kDa)
250 –
150 –
100 –
75 –
Vinculin (114 kDa)

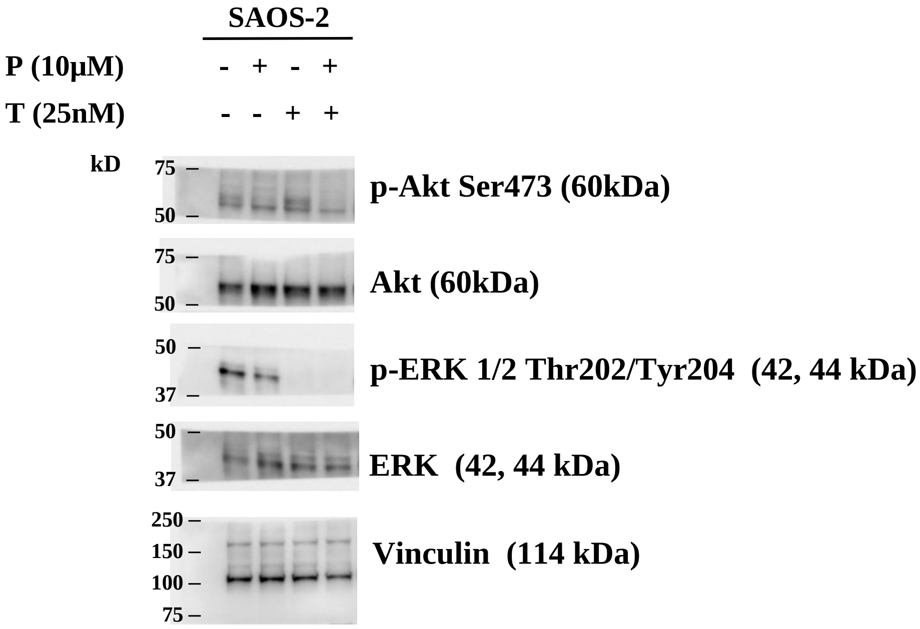
SAOS-2
P (10µM) - + - +
T (25nM) - - + +
kD
75
50
p-Akt Ser473 (60kDa)
75
50
Akt (60kDa)
50
37
p-ERK 1/2 Thr202/Tyr204 (42, 44 kDa)
50
37
ERK (42, 44 kDa)
250
150
100
75
Vinculin (114 kDa)

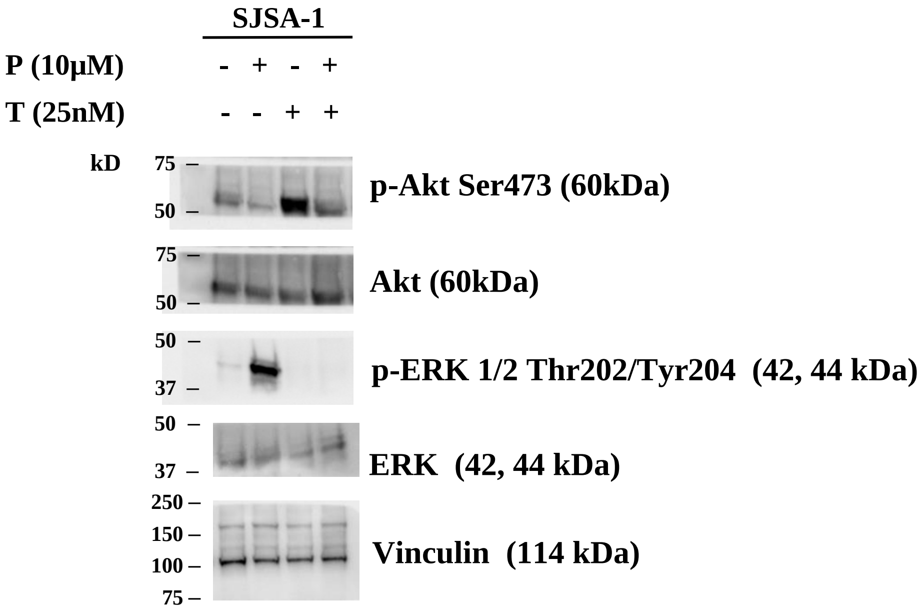
SJSA-1
P (10µM) - + - +
T (25nM) - - + +
kD
75
50
p-Akt Ser473 (60kDa)
75
50
Akt (60kDa)
50
37
p-ERK 1/2 Thr202/Tyr204 (42, 44 kDa)
50
37
ERK (42, 44 kDa)
250
150
100
75
Vinculin (114 kDa)

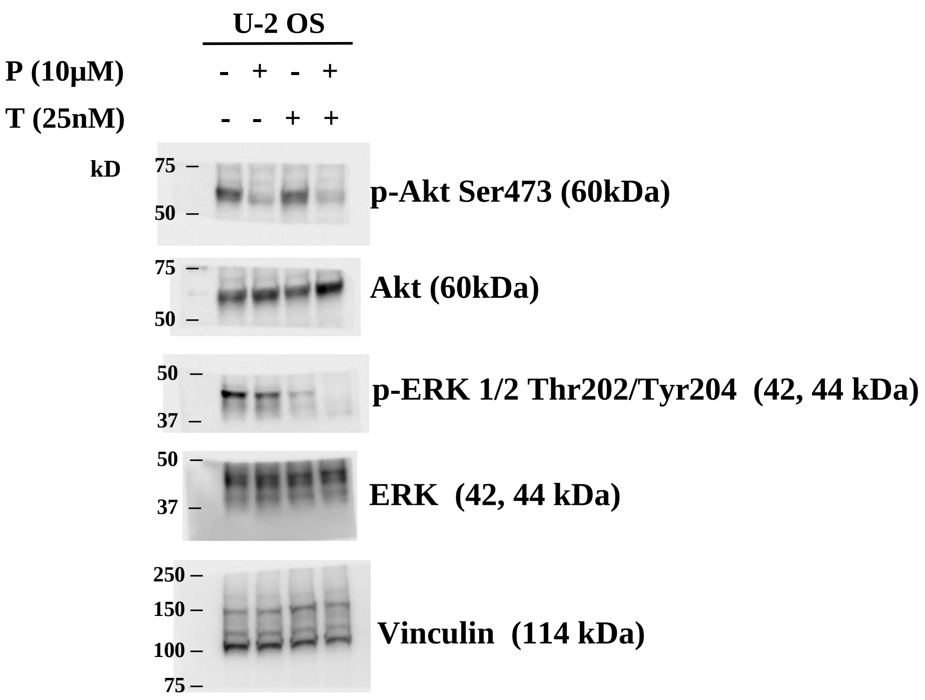
U-2 OS
P (10μM) - + - +
T (25nM) - - + +
kD
75
50
p-Akt Ser473 (60kDa)
75
50
Akt (60kDa)
50
37
p-ERK 1/2 Thr202/Tyr204 (42, 44 kDa)
50
37
ERK (42, 44 kDa)
250
150
100
75
Vinculin (114 kDa)

Figure 3 E

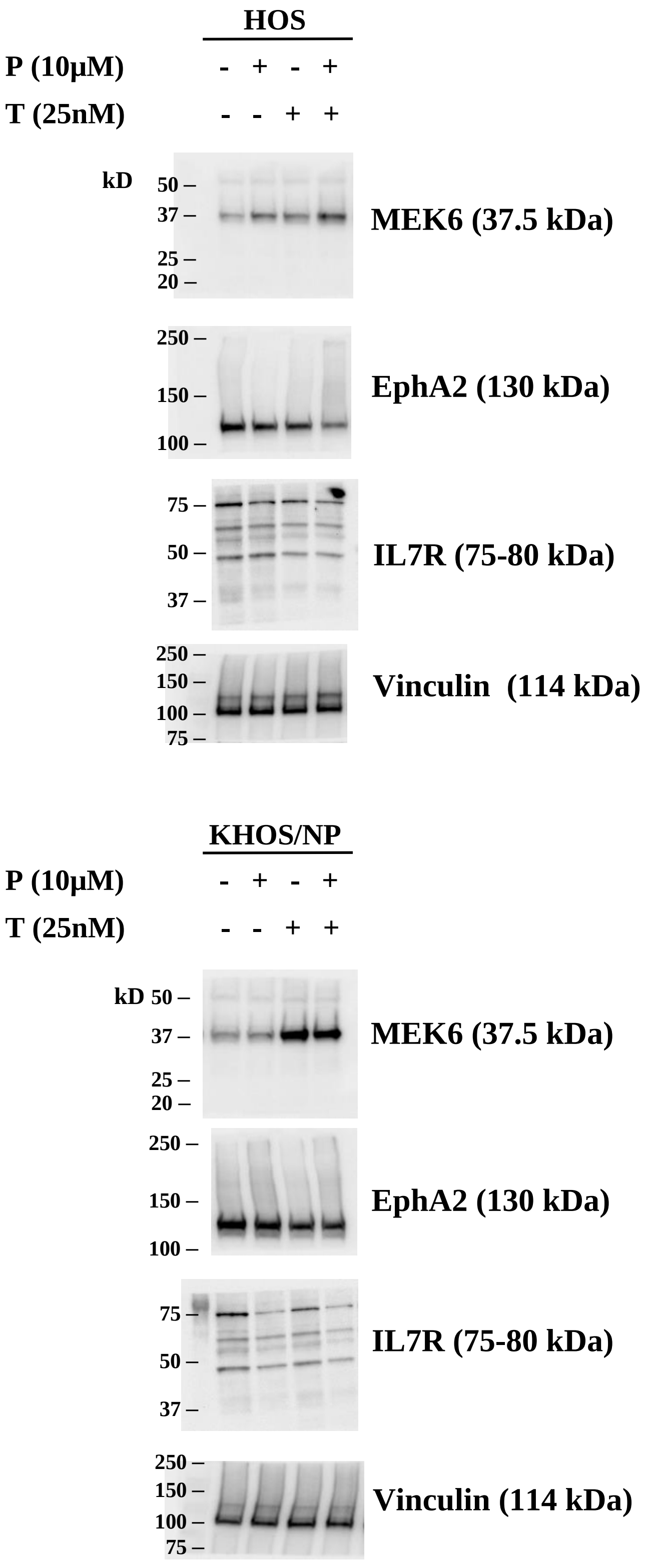
HOS
P (10µM) - + - +
T (25nM) - - + +
kD
50
37
25
20
MEK6 (37.5 kDa)
250
150
100
EphA2 (130 kDa)
75
50
37
IL7R (75-80 kDa)
250
150
100
75
Vinculin (114 kDa)
KHOS/NP
P (10µM) - + - +
T (25nM) - - + +
kD
50
37
25
20
MEK6 (37.5 kDa)
250
150
100
EphA2 (130 kDa)
75
50
37
IL7R (75-80 kDa)
250
150
100
75
Vinculin (114 kDa)

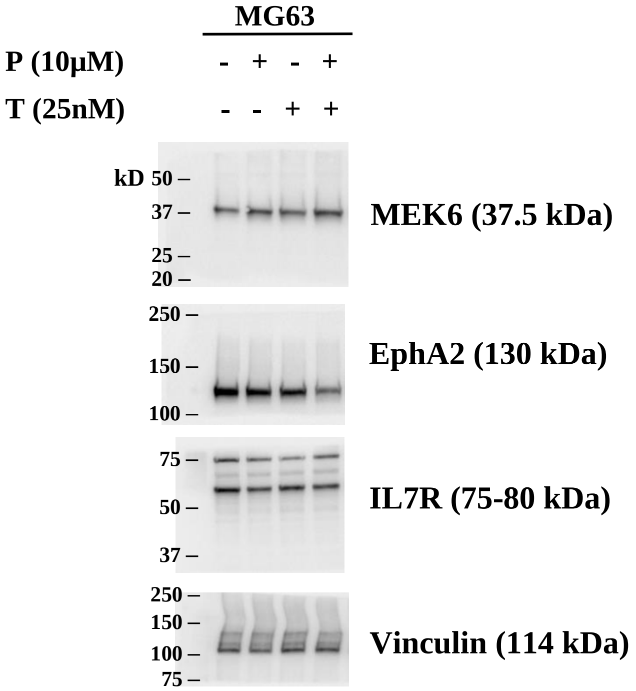
MG63
P (10μM) - + - +
T (25nM) - - + +
kD 50 –
37 –
25 –
20 –
MEK6 (37.5 kDa)
250 –
150 –
100 –
EphA2 (130 kDa)
75 –
50 –
37 –
IL7R (75-80 kDa)
250 –
150 –
100 –
75 –
Vinculin (114 kDa)

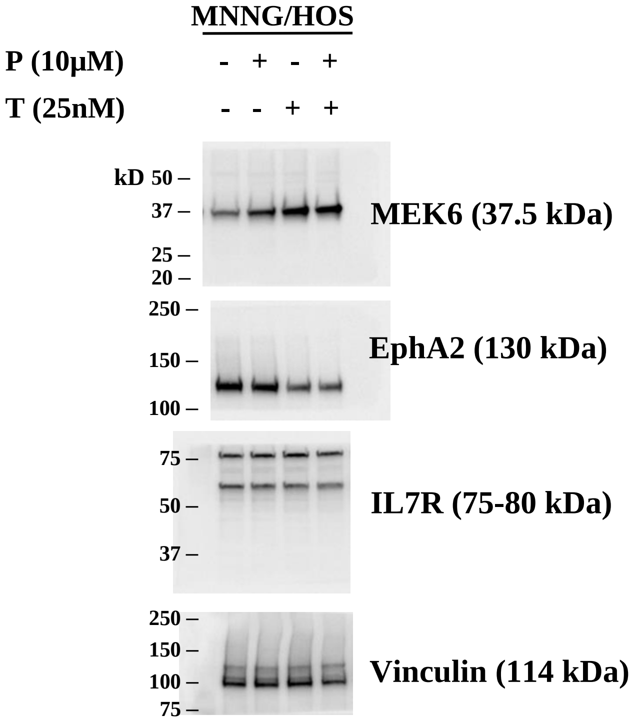
MNNG/HOS
P (10μM) - + - +
T (25nM) - - + +
kD 50 –
37 –
25 –
20 –
MEK6 (37.5 kDa)
250 –
150 –
100 –
EphA2 (130 kDa)
75 –
50 –
37 –
IL7R (75-80 kDa)
250 –
150 –
100 –
75 –
Vinculin (114 kDa)

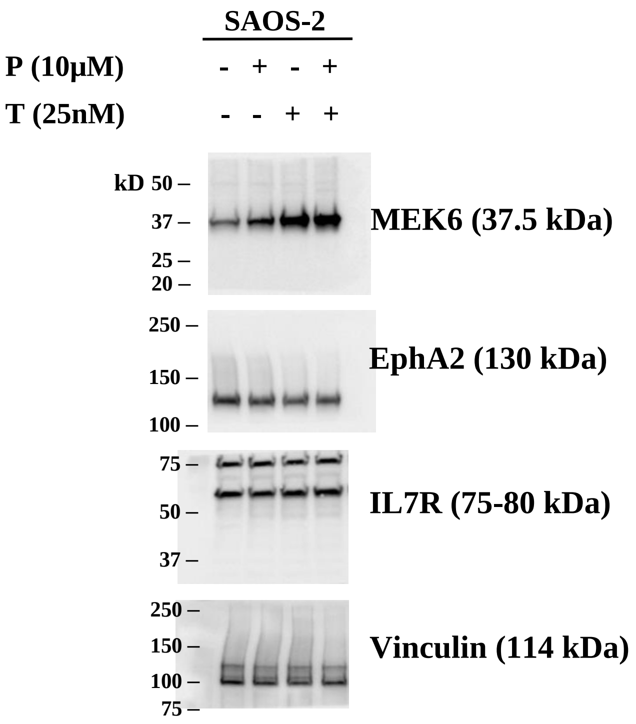
SAOS-2
P (10μM) - + - +
T (25nM) - - + +
kD 50 –
37 –
25 –
20 –
MEK6 (37.5 kDa)
250 –
150 –
100 –
EphA2 (130 kDa)
75 –
50 –
37 –
IL7R (75-80 kDa)
250 –
150 –
100 –
75 –
Vinculin (114 kDa)

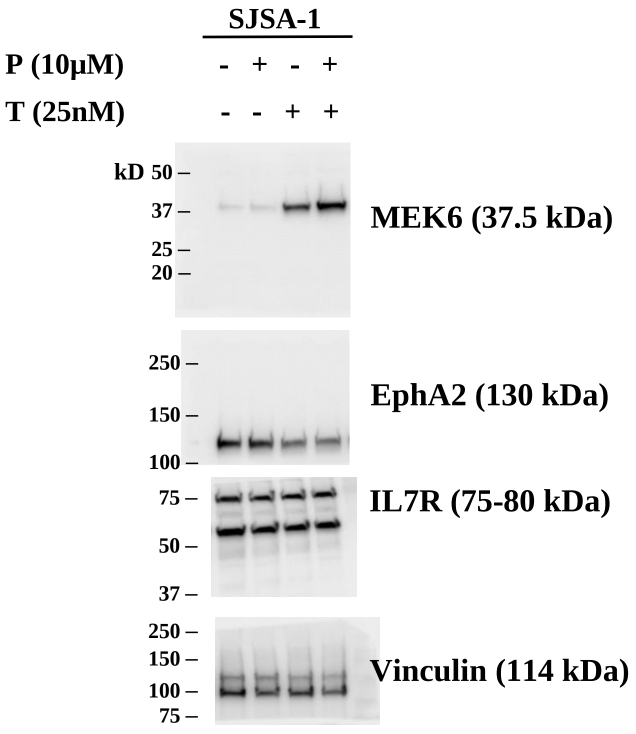
SJSA-1
P (10μM) - + - +
T (25nM) - - + +
kD 50 –
37 –
25 –
20 –
MEK6 (37.5 kDa)
250 –
150 –
100 –
EphA2 (130 kDa)
75 –
50 –
37 –
IL7R (75-80 kDa)
250 –
150 –
100 –
75 –
Vinculin (114 kDa)

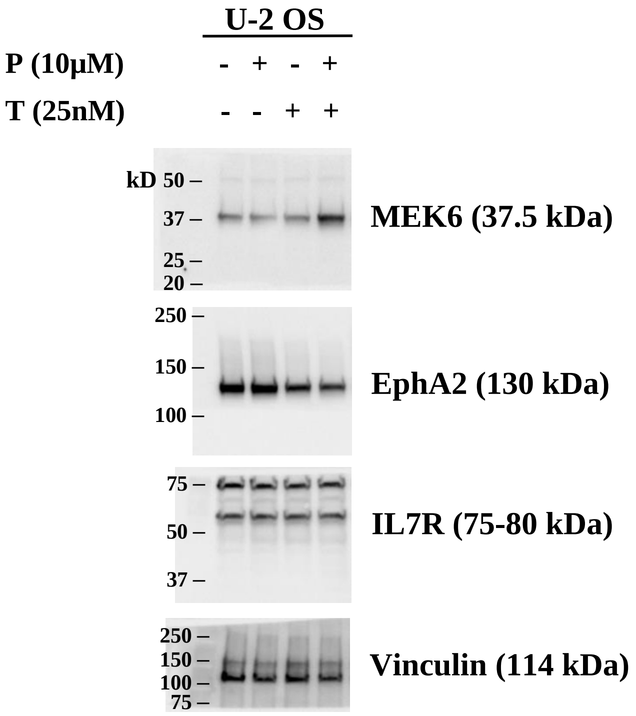
U-2 OS
P (10μM) - + - +
T (25nM) - - + +
kD 50 –
37 –
25 –
20 –
MEK6 (37.5 kDa)
250 –
150 –
100 –
EphA2 (130 kDa)
75 –
50 –
37 –
IL7R (75-80 kDa)
250 –
150 –
100 –
75 –
Vinculin (114 kDa)

Figure 4 A

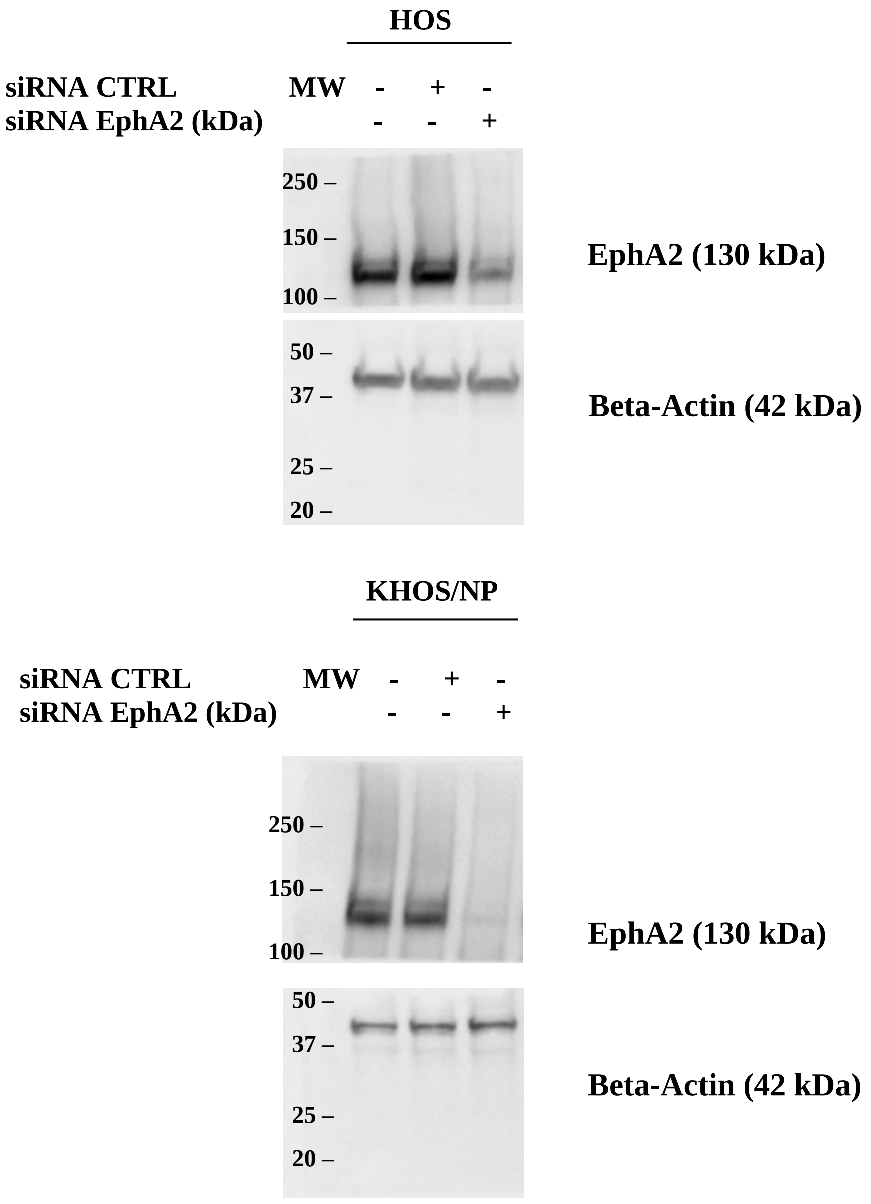
HOS
siRNA CTRL
siRNA EphA2 (kDa)
MW
- + -
- - +
250 –
150 –
100 –
EphA2 (130 kDa)
50 –
37 –
25 –
20 –
Beta-Actin (42 kDa)
KHOS/NP
siRNA CTRL
siRNA EphA2 (kDa)
MW
- + -
- - +
250 –
150 –
100 –
EphA2 (130 kDa)
50 –
37 –
25 –
20 –
Beta-Actin (42 kDa)

Figure 5 A

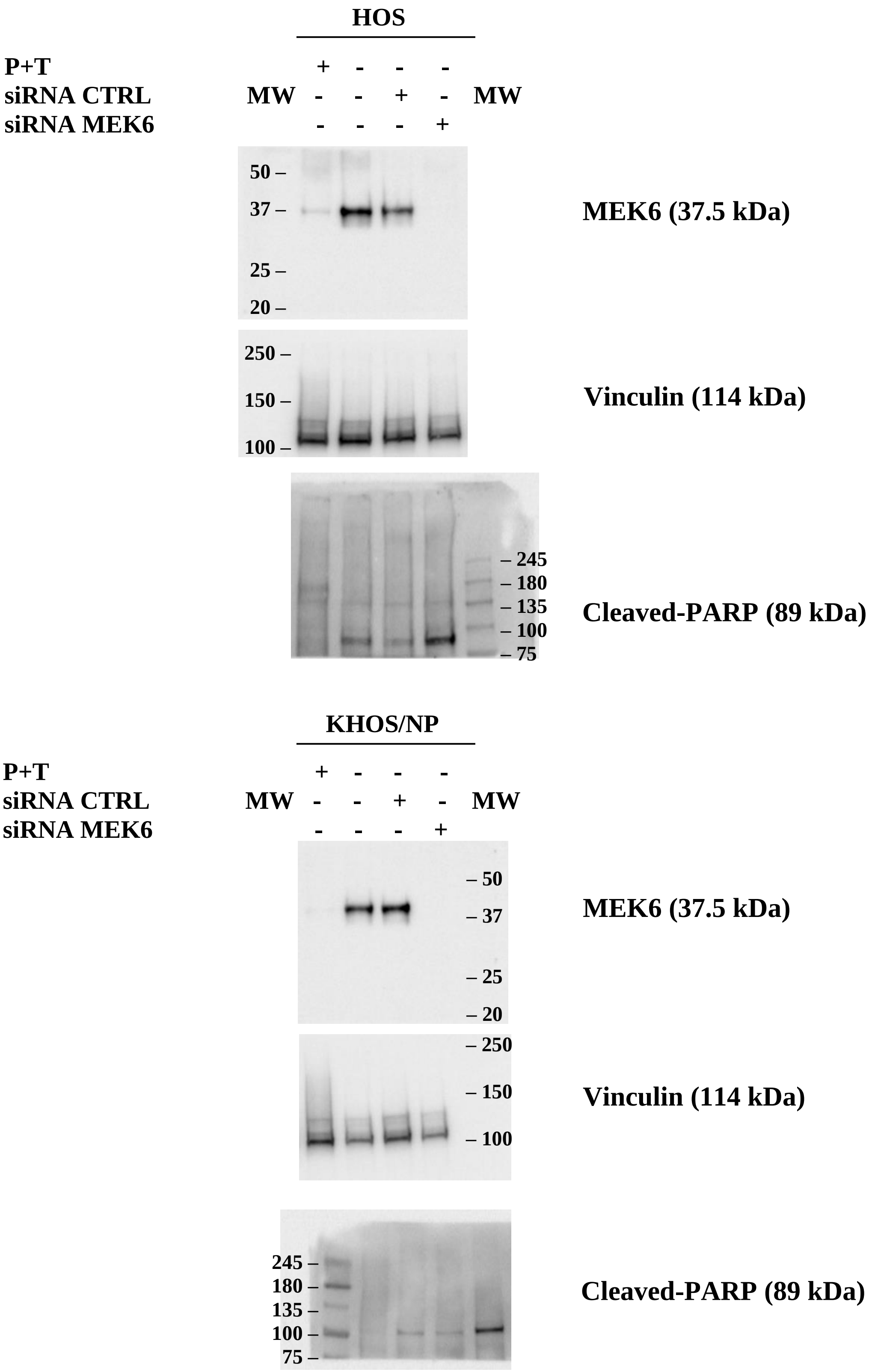
HOS
P+T
siRNA CTRL
siRNA MEK6
MW
+ - - -
- - + -
- - - +
MW
50 –
37 –
25 –
20 –
MEK6 (37.5 kDa)
250 –
150 –
100 –
Vinculin (114 kDa)
– 245
– 180
– 135
– 100
– 75
Cleaved-PARP (89 kDa)
KHOS/NP
P+T
siRNA CTRL
siRNA MEK6
MW
+ - - -
- - + -
- - - +
MW
– 50
– 37
– 25
– 20
MEK6 (37.5 kDa)
– 250
– 150
– 100
Vinculin (114 kDa)
245 –
180 –
135 –
100 –
75 –
Cleaved-PARP (89 kDa)

Figure 5 I

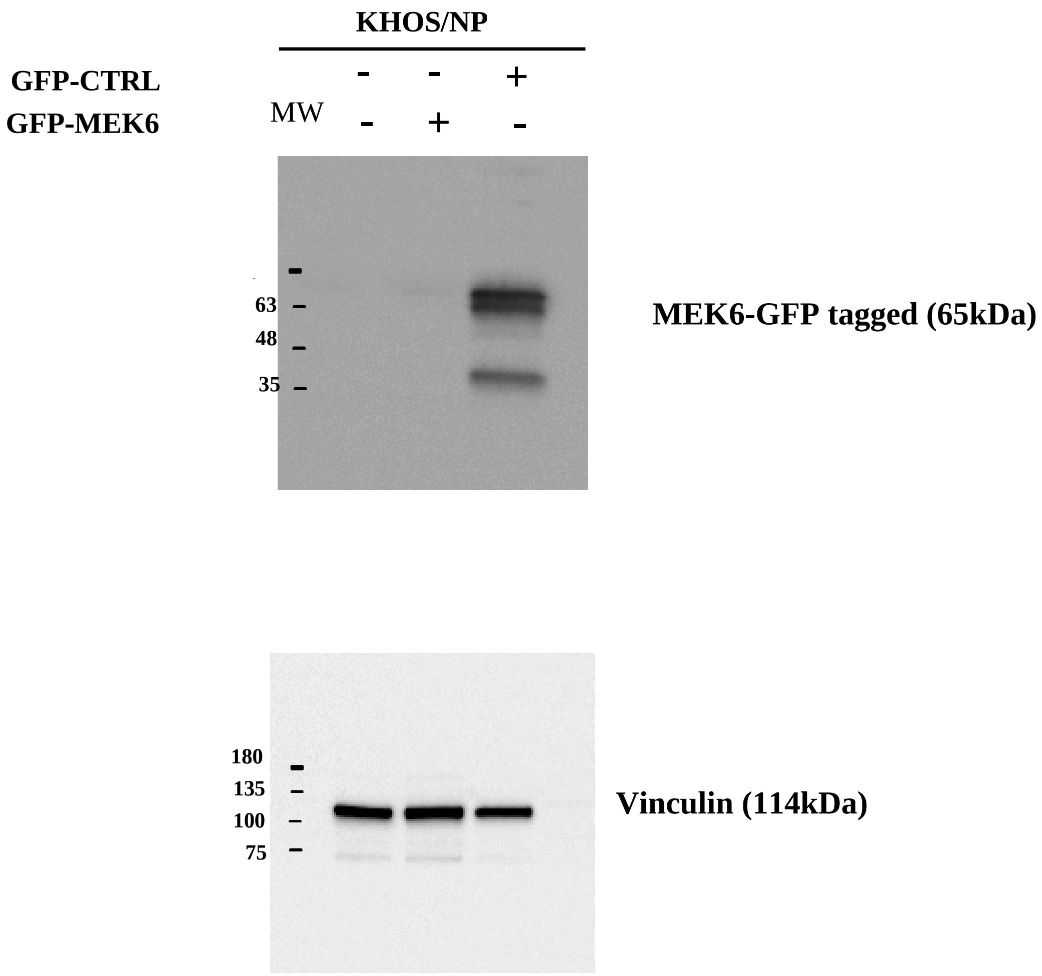
KHOS/NP
GFP-CTRL
GFP-MEK6
MW
- - +
- + -
63
48
35
MEK6-GFP tagged (65kDa)
180
135
100
75
Vinculin (114kDa)

Figure S2

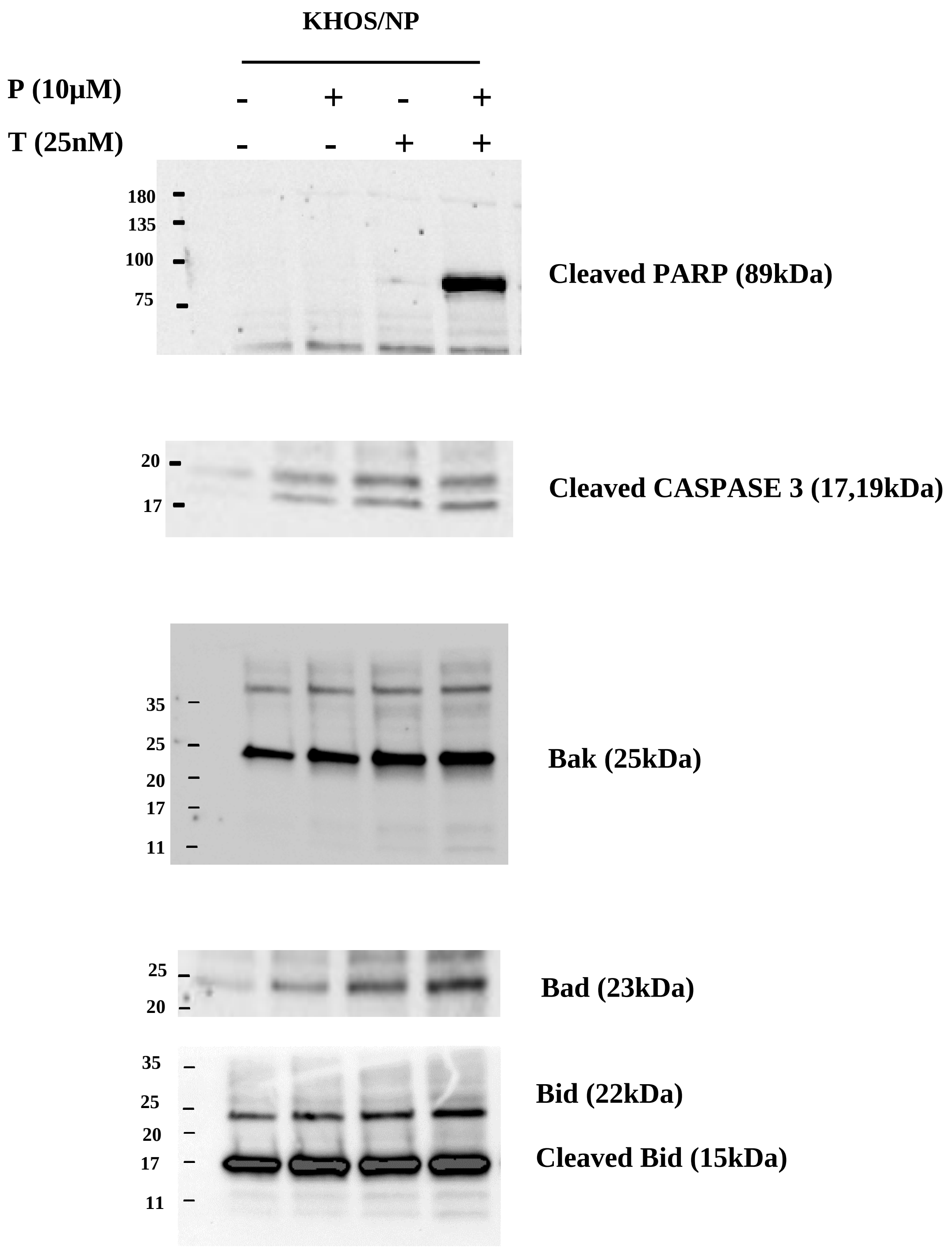
KHOS/NP
P (10μM) - + - +
T (25nM) - - + +
180
135
100
75
Cleaved PARP (89kDa)
20
17
Cleaved CASPASE 3 (17,19kDa)
35
25
20
17
11
Bak (25kDa)
25
20
Bad (23kDa)
35
25
20
17
11
Bid (22kDa)
Cleaved Bid (15kDa)

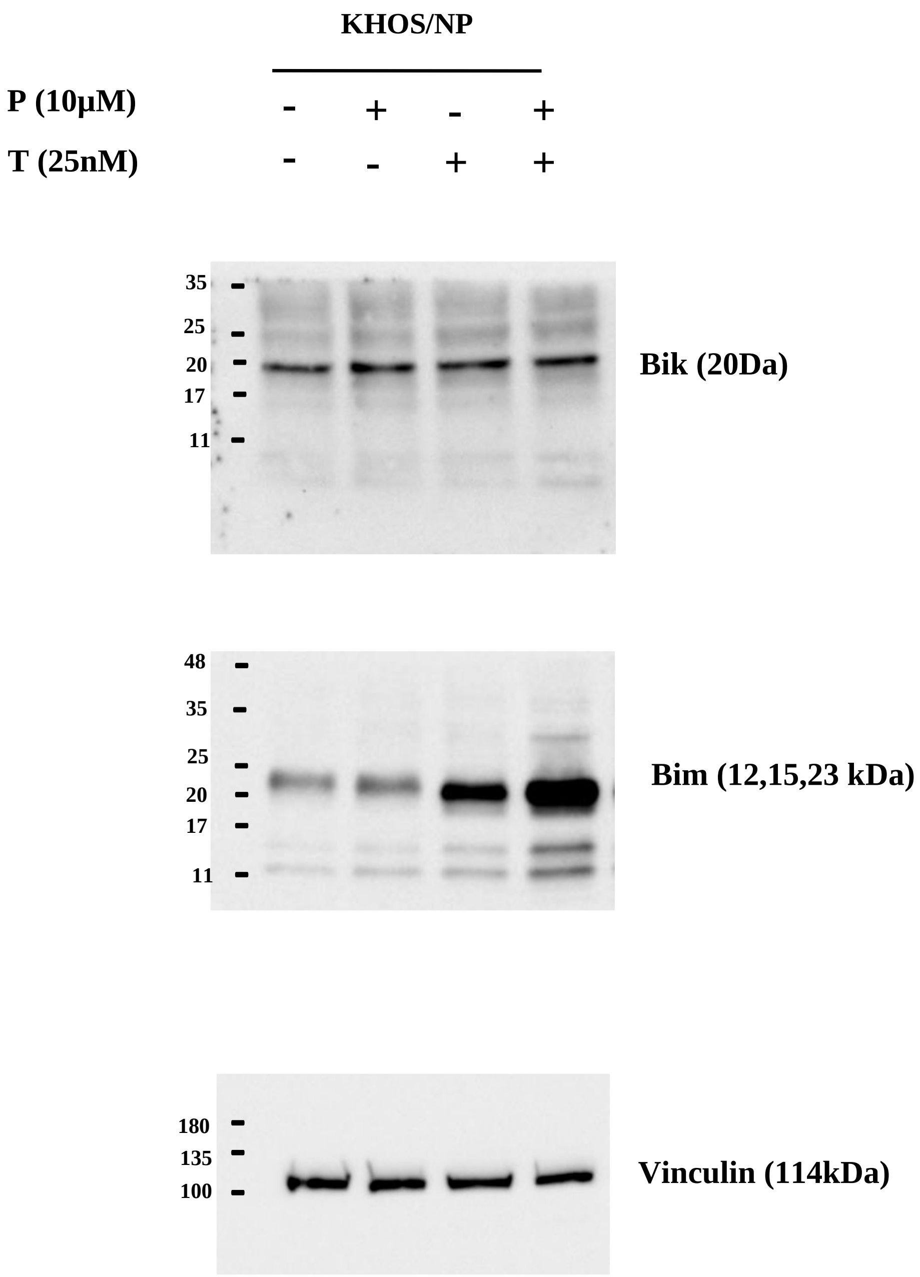
KHOS/NP
P (10µM) - + - +
T (25nM) - - + +
35
25
20
17
11
Bik (20Da)
48
35
25
20
17
11
Bim (12,15,23 kDa)
180
135
100
Vinculin (114kDa)

Figure S3

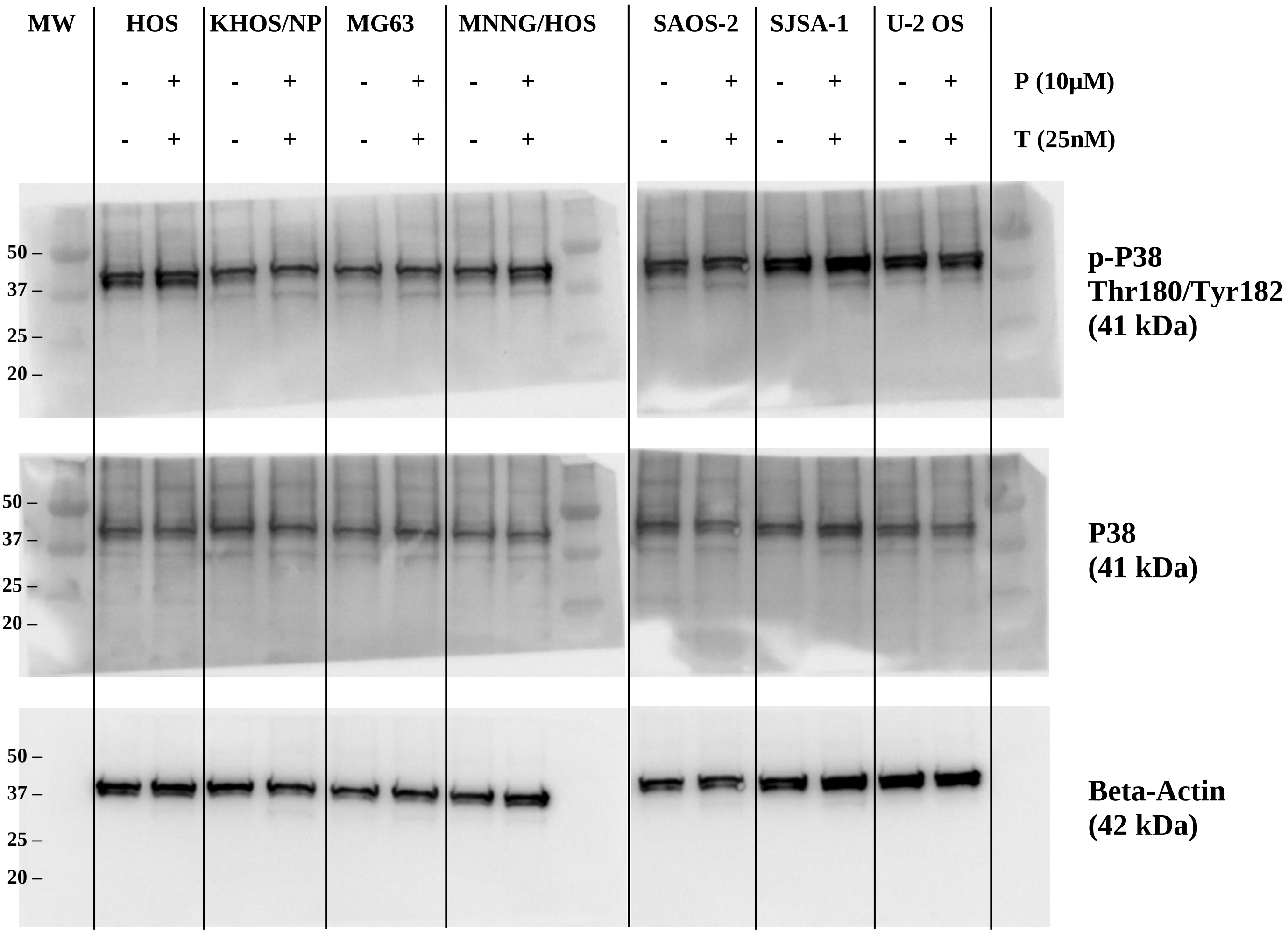
MW
HOS
KHOS/NP
MG63
MNNG/HOS
SAOS-2
SJSA-1
U-2 OS
P (10μM)
T (25nM)
50 –
37 –
25 –
20 –
p-P38 Thr180/Tyr182 (41 kDa)
P38 (41 kDa)
Beta-Actin (42 kDa)
